# Regulatory effects of salt–nitrogen interaction on the growth and photosynthetic characteristics of *Limonium bicolor* in the Yellow River Delta

**DOI:** 10.3389/fpls.2026.1940663

**Published:** 2026-09-08

**Authors:** Lechong Nie, Wenji Zhang, Yao Sun, Mengxue Xing, Xi Jia, Lu Wang, Dengchao Gao, Pengshuai Shao, Jingkuan Sun, Jinzhao Ma

**Affiliations:** 1 Shandong Key Laboratory of Eco-Environmental Science for the Yellow River Delta, Shandong University of Aeronautics, Binzhou, China; 2 Yantai Agricultural Extension Center, Yantai, China; 3 Institute of Modern Agriculture on Yellow River Delta, Shandong Academy of Agricultural Sciences, Dongying, China; 4 Wudi Land Consolidation and Reserve Center, Binzhou, China

**Keywords:** ecological stoichiometry, *Limonium bicolor*, photosynthetic physiology, salt–nitrogen interaction, yellow river delta

## Abstract

The Yellow River Delta, as a typical coastal saline soil distribution area in northern China, also faces increasingly prominent nitrogen input problems. To investigate the effects of salt–nitrogen interaction on the growth and photosynthetic physiology of the typical halophyte *Limonium bicolor* in the Yellow River Delta, this study established a pot-controlled experiment with three salt gradients (no salt addition, 0.3%, and 0.6% salinity) and four nitrogen addition levels (0, 15, 30, and 45 kg·N·ha^−1^·yr^−1^). Growth parameters, photosynthetic characteristics, element contents, and soil physicochemical properties of *L. bicolor* were measured. The results showed that salt and nitrogen addition had significant interactive effects on plant height, biomass, net photosynthetic rate, and other indicators. Compared with the no-salt and 0 kg·N·ha^−1^·yr^−1^ treatment, the treatment combining 0.6% salinity and 45 kg·N·ha^−1^·yr^−1^ significantly increased plant height, aboveground biomass, and net photosynthetic rate. Nitrogen addition increased the net photosynthetic rate, stomatal conductance, SPAD value, and total chlorophyll content of *L. bicolor* in a saline environment. Salt–nitrogen interaction significantly regulated total nitrogen in aboveground and belowground parts, and total phosphorus in belowground parts, of *L. bicolor*. Salt significantly increased soil pH, electrical conductivity, and organic matter content. Foliar nitrogen input, accompanied by plant–soil feedback processes, altered certain soil nutrient indices, with soil total nitrogen and inorganic nitrogen contents showing significant responses; however, it should be noted that long-term high nitrogen input may drive a shift toward phosphorus limitation. In ecological restoration practices, coordinated management of nitrogen and phosphorus should be given due attention. Structural equation modeling indicated that nitrogen addition was the primary exogenous factor driving *L. bicolor* growth. In conclusion, moderate salt–nitrogen interaction (i.e., the 0.6% salinity and 45 kg·N·ha^−1^·yr^−1^ treatment) synergistically promoted the growth and photosynthesis of *L. bicolor*, although higher nitrogen addition may induce a potential nitrogen saturation-like response.

## 1 Introduction

The Yellow River Delta is situated in the coastal area along the mouth of the Yellow River, south of the Bohai Sea. As a typical distribution zone of coastal saline soils, it represents one of the major concentrated areas of salt-affected soils in China. Saline soil formation in the Yellow River Delta results from multiple interacting factors, with a shallow water table and highly mineralized groundwater being the primary drivers (Zhao et al., 2026; Zhou et al., 2026). In addition, anthropogenic reclamation, seawater intrusion, and climate change have further exacerbated soil salinization, severely constraining the sustainable development of the local ecosystems (Wang et al., 2025). In the Yellow River Delta, saline soils account for over 50% of the total area, with NaCl being the predominant salt component (Sun et al., 2023), and they are typified by high Na^+^ content and low fertility (Xia et al., 2019). The surface soil salt content in this region exhibits strong spatial heterogeneity, generally ranging from 0.08% to 1.36%, with lightly and moderately salinized soils being the most widely distributed types (Fu et al., 2020). Excessive soil salinity disrupts the osmotic balance between plant cells and their external environment, induces ion toxicity, limits water uptake, and suppresses photosynthetic rates, thereby retarding plant growth and development (Ahmed et al., 2021). Furthermore, soil salinity influences key nitrogen transformation processes, including hydrolysis, volatilization, and nitrification (Tao and Liu, 2024), rendering the behavior of nitrogen in saline soils more complex.

Since the Industrial Revolution, global nitrogen deposition has risen dramatically, with increases of more than threefold in some regions (Cen et al., 2025). Currently, average annual nitrogen deposition in many parts of the world already exceeds 10 kg·ha^−1^·yr^−1^, and is projected to reach roughly twice pre-industrial levels by around 2050 (Andersson et al., 2025). The Yellow River Delta has emerged as one of China’s regions with the highest nitrogen deposition, facing increasingly severe nitrogen input pressures (Guan et al., 2019). Nitrogen is an essential component of proteins, nucleic acids, chlorophyll, and secondary metabolites; it is not only an essential nutrient for plant growth but also a key factor limiting photosynthesis and overall plant development in the Yellow River Delta (Ahanger et al., 2019). Appropriate nitrogen application has been shown to significantly increase leaf chlorophyll content and photosynthetic rates, thereby alleviating salt-induced photosynthetic inhibition; however, excessive nitrogen supply can suppress photosynthetic physiology, reduce light-use and nitrogen-use efficiencies, and retard biomass accumulation (Song et al., 2019). The interaction between salinity and nitrogen profoundly influences plant nitrogen metabolism and salt stress response. While nitrogen addition can mitigate salt-induced damage to plants, over-application may exacerbate soil salinization, inhibit seed germination, and intensify the adverse effects of salt stress on plant performance (Xu et al., 2025).

The coastal saline-alkaline lands of the Yellow River Delta support a variety of salt-tolerant plant species, which play critical roles in maintaining wetland ecological functions, improving soil structure, and promoting nutrient cycling (Li et al., 2026). *Limonium bicolor*, a perennial herbaceous species belonging to the genus *Limonium* in the family Plumbaginaceae, is widely distributed along the coastal areas of the Bohai Bay in China (Yin et al., 2018). The dominant halophytes in this region include *L. bicolor*, *Suaeda salsa*, and *Phragmites australis*, which collectively contribute to maintaining vegetation stability and ecosystem functioning in coastal wetlands (Li et al., 2021). Unlike most salt-tolerant plants that rely on salt exclusion or dilution strategies, *L. bicolor* is a typical recretohalophyte, characterized by multicellular salt glands distributed on its leaves and stems. These glands actively secrete excess Na^+^ out of the plant body, thereby effectively alleviating salt stress and maintaining cellular ion homeostasis (Zhao et al., 2023a; Zhu et al., 2025). Owing to this unique salt-tolerance mechanism, *L. bicolor* is recognized as a pioneer species for the amelioration of saline-alkali soils (Sun et al., 2025). In addition, it possesses ornamental and medicinal values, making it an ideal candidate material that integrates ecological restoration, resource utilization, and scientific research (Meng et al., 2024).

However, existing studies on salt–nitrogen interactions have largely focused on halophytes such as *S. salsa* and *P. australis* (Gong et al., 2022; Wang et al., 2022), whereas research on *L. bicolor* has predominantly examined the effects of single salinity stress (Yin et al., 2024; Zhu et al., 2024). Consequently, the synergistic or antagonistic effects of combined salt and nitrogen addition on this species remain poorly understood. In particular, against the backdrop of intensifying global nitrogen deposition, a systematic understanding of how exogenous nitrogen input affects the growth, photosynthetic processes, and elemental allocation of *L. bicolor* in saline environments is still lacking. Therefore, in this study, we used *L. bicolor* as the experimental subject and established varying salinity gradients and nitrogen addition levels to systematically investigate the effects of salt–nitrogen interaction on the growth performance, photosynthetic characteristics, and elemental allocation of this species. The aim was to elucidate the adaptive mechanisms of *L. bicolor* in response to future environmental changes, and to provide a scientific basis for the management of exogenous nitrogen input and nutrient regulation of halophytes in the ecological restoration of saline-alkali lands in the Yellow River Delta.

## 2 Materials and methods

### 2.1 Test materials

A pot experiment was conducted in the intelligent greenhouse at the Shandong Provincial Key Laboratory of Eco-Environmental Science for the Yellow River Delta, located in Binzhou City, Shandong Province, which lies in the core area of the Yellow River Delta. During the experiment, the day/night temperature was maintained at 25 ± 2 °C/18 ± 2 °C via an environmental control system, with relative humidity controlled at 60 ± 10%. The test soil was non-saline fluvo-aquic soil collected from the Yellow River floodplain in the Yellow River Delta. Its basic physicochemical properties were determined as follows: pH 7.10 ± 0.30, electrical conductivity (EC) 7.62 ± 0.20 mS·cm^−1^, salt content 1.33 ± 0.35 g·kg^−1^, organic matter content 12.50 ± 1.20 g·kg^−1^, total nitrogen (TN) 0.43 ± 0.03 g·kg^−1^, and total phosphorus (TP) 0.54 ± 0.02 g·kg^−1^.

Seeds of *L. bicolor* were collected from the natural distribution area in the Yellow River Delta, Dongying City, Shandong Province, China (N38°1′18″, E118°44′2″). The study region has a warm-temperate monsoon climate, with a mean annual temperature of 12.6 °C and mean annual precipitation ranging from 550 to 640 mm (Zhang et al., 2023). The physicochemical properties of the surface soil at this site were as follows: pH 7.83, EC 5.38 mS·cm^−1^, organic matter 16.2 g·kg^−1^, and TN 0.32 g·kg^−1^. The vegetation coverage at the sampling site ranged from approximately 40% to 60%, with the dominant species including *L. bicolor*, *S. salsa*, and *P. australis*. In October 2023, seeds were collected from mature, healthy mother plants without pests or diseases within a 100 m × 100 m plot. After collection, the seeds were thoroughly air-dried and stored at 4 °C until further use.

### 2.2 Experimental design

The experiment employed a two-factor completely randomized design, with salinity and nitrogen addition levels set based on the ecological realities of saline-alkali soils in the Yellow River Delta. Three salinity gradients were established, covering a typical range from non-salinized to moderately salinized conditions in this region: S_0_ (no salt addition), S_1_ (soil salt content of 0.3%, lightly salinized), and S_2_ (soil salt content of 0.6%, moderately salinized). Taking into account the increasing trend of regional nitrogen inputs and referencing previous nitrogen addition experiments (Wang et al., 2021), four nitrogen addition levels were set: N_0_ (0 kg·N·ha^−1^·yr^−1^), N_15_ (15 kg·N·ha^−1^·yr^−1^), N_30_ (30 kg·N·ha^−1^·yr^−1^), and N_45_ (45 kg·N·ha^−1^·yr^−1^). In total, there were 12 treatments, with five replicates, resulting in 60 pots.

The experiment was conducted from May to October 2024. Full and plump seeds of *L. bicolor* were selected and surface-sterilized sequentially: immersed in 75% ethanol for 5 min and 6% NaClO solution for 15 min. After sterilization, the seeds were rinsed 3–5 times with sterile water, blotted dry on sterile filter paper, and then sown in seedling substrate (Gao et al., 2021). After germination and seedling establishment, the seedlings were transplanted into plastic pots (upper diameter 32 cm, bottom diameter 30 cm, height 35 cm), each filled with 3.5 kg of soil. Following a 14-day acclimation period, seedlings were thinned to five healthy individuals per pot. Salinity was imposed by adding NaCl solution, with target soil salt contents achieved through repeated quantitative irrigation during the acclimation period. In this study, foliar application of urea solution was adopted as the nitrogen input method, which allows precise control over the timing and dosage of nitrogen supply; moreover, urea can be rapidly absorbed through leaf stomata and cuticles upon direct foliar contact (Liu et al., 2021). From June to August during the growing season, urea solutions at concentrations of 0.5%, 1.0%, and 1.5% were sprayed on the leaves on five separate occasions in the early evening on clear days (Uscola et al., 2014), with the control receiving an equivalent volume of deionized water. The interval between consecutive applications was approximately 15 days, and the spray volume was approximately 130 mL·m^−^² per application. All other management practices were kept consistent across treatments throughout the experimental period.

### 2.3 Sample collection and analysis

#### 2.3.1 Leaf photosynthetic parameters

On clear days in September 2024, healthy functional leaves of *L. bicolor* from each treatment were selected, and photosynthetic parameters were measured using a Li-6400XT portable photosynthesis system (LI-COR Inc., Lincoln, NE, USA). During the measurement, the light intensity was set at 1000 μmol m^−^² s^−1^ (Zhang et al., 2016), and the leaf chamber temperature was maintained at 25 °C. The net photosynthetic rate (*P*_n_), stomatal conductance (*G*_s_), intercellular CO_2_ concentration (*C*_i_), transpiration rate (*T*_r_), and the ambient CO_2_ concentration ratio (*C*_a_) (Peng et al., 2025)were recorded. The stomatal limitation value (*L*_s_) and water use efficiency (WUE) were calculated using the following formulas (Renjie et al., 2025): $L_{s}=1-C_{i}/C_{a}$; $WUE=P_{n}/T_{r}$.

The SPAD value was measured on the same functional leaves using a SPAD-502 chlorophyll meter, taking three measurements while avoiding the main vein and averaging the values. At the same time, healthy leaves of *L. bicolor* were collected and brought to the laboratory. Fresh leaf samples of 0.2 g were weighed, and pigments were extracted using the 95% ethanol immersion method (Sun et al., 2021) (immersed in darkness for 24 h until the leaves turned white). The absorbance of the extract was measured at 665, 649, and 470 nm using a spectrophotometer. The contents of chlorophyll a (Chl a), chlorophyll b (Chl b), carotenoids (Car), and total chlorophyll (Chl a+b) were calculated according to the formulas (Chen et al., 2025):


$$\text{Chl a }\left(\text{mg}\cdot \text{g}-1\right)=\left(13.95\times {\text{A}}_{665}-6.88\times {\text{A}}_{649}\right)\times \frac{V}{W\times 1000}$$

$$\text{Chl b }\left(\text{mg}\cdot \text{g}-1\right)=\left(24.96\times {\text{A}}_{649}-7.32\times {\text{A}}_{665}\right)\times \frac{V}{W\times 1000}$$

$$\text{Car }\left(\text{mg}\cdot \text{g}-1\right)=\frac{\left(1000\times {\text{A}}_{470}-2.05\times \text{Ca}-114.8\times \text{Cb}\right)}{245}\times \frac{V}{W\times 1000}$$

Chl a+b (mg·g−1)=Chl a+Chl b

In the formulas, A_665_, A_649_, and A_470_ represent the absorbance values at wavelengths of 665, 649, and 470 nm, respectively; V is the volume of the extraction solution (mL); and W is the fresh weight of the leaves (g).

#### 2.3.2 Plant growth indicators

At the end of the growing season of *L. bicolor* in October 2024, five representative plants were randomly selected from each treatment group, and the roots were carefully washed clean. Plant height and main root length were measured with a tape measure, and stem base diameter was measured with a vernier caliper. Subsequently, the plants were divided into aboveground parts (stems and leaves) and belowground parts (roots), placed separately into kraft paper bags, oven-dried at 105 °C for 30 minutes to deactivate enzymes, and then dried at 75 °C to constant weight, after which the biomass was recorded.

#### 2.3.3 Plant element contents

The dried plant samples were ground and passed through a sieve. Total carbon (TC) and TN were determined using an elemental analyzer (vario MACRO CUBE, Elementar, Germany). After digestion of the plant samples with sulfuric acid–hydrogen peroxide, TP was determined by the molybdenum–antimony ascorbic acid colorimetric method, and total potassium (TK) and total sodium (TNa) contents were determined using an atomic absorption spectrophotometer (AA-6800, Shimadzu, Japan) (Zhang et al., 2022). The C:N, C:P, N:P ratios, and the K/Na ratio were calculated.

#### 2.3.4 Soil physicochemical properties

Soil samples were collected during the same period. Fresh soil samples were passed through a sieve, and then the nitrate nitrogen (NO_3_^−^-N) and ammonium nitrogen (NH_4_^+^-N) contents were determined using an AA3 continuous flow injection analyzer (SEAL Analytical GmbH, Norderstedt, Germany) after potassium chloride extraction. Soil microbial biomass carbon (MBC) and nitrogen (MBN) were determined using the chloroform fumigation–potassium sulfate extraction method and measured with a total organic carbon analyzer (enviro TOC, Elementar, Germany). Additional air-dried soil samples were sieved for the determination of the following indicators: pH was measured using a PHS-3C precision pH meter, and EC was measured using a conductivity meter; Available phosphorus (AP) was determined by the sodium bicarbonate extraction–molybdenum–antimony ascorbic acid colorimetric method; Available potassium (AK) was determined by the ammonium acetate extraction–flame photometric method. Soil organic matter (SOM) was determined by the potassium dichromate–external heating method. TC and TN were determined using an elemental analyzer (vario MACRO CUBE, Elementar, Germany), and TP was determined by the molybdenum–antimony ascorbic acid colorimetric method after digestion with sulfuric acid–perchloric acid (Bai et al., 2025). The ecological stoichiometric ratios of the soil were calculated.

### 2.4 Data analysis

Experimental data were collated using Microsoft Office Excel 2019. Two-way analysis of variance (ANOVA) for significance testing was performed using IBM SPSS Statistics 27. Graphs were generated using Origin 2024. Correlation heatmaps were constructed using R language. Structural equation modeling (SEM) was established, and path coefficients were estimated using the maximum likelihood estimation method.

## 3 Results and analysis

### 3.1 Effects of salt–nitrogen addition on growth characteristics of *L. bicolor*

**Figure 1:**
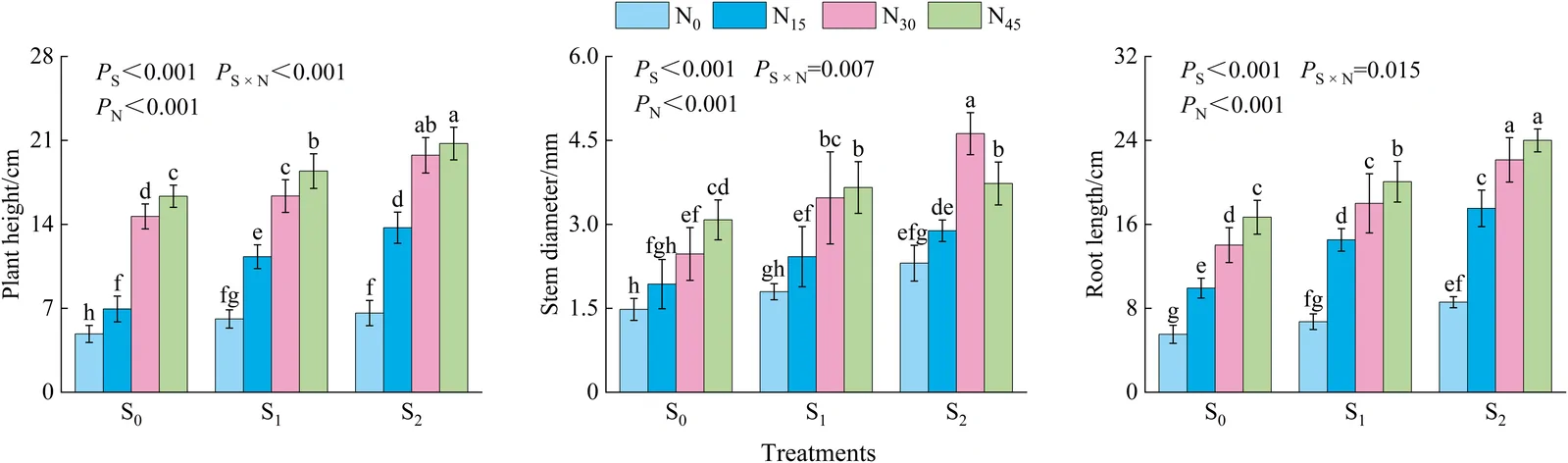
Effects of soil salinity and nitrogen addition on growth indicators of *L. bicolor*. S_0_–S_2_ represent soil salt contents of blank control, 0.3%, and 0.6%, respectively; N_0_–N_45_ represent nitrogen addition levels of 0, 15, 30, and 45 kg·N·ha^−1^·yr^−1^, respectively; S denotes salt gradient, N denotes nitrogen addition level, and S×N denotes the combined application of salt and nitrogen; different lowercase letters indicate significant differences among treatments (*P* < 0.05).

Different salt and nitrogen treatments had significant effects on plant height, stem diameter, and root length of *L. bicolor*, and the interaction between salt and nitrogen addition was also significant (**Figure 1**). Under the S_1_ gradient, plant height and root length in the N_45_ treatment were significantly higher than those in the N_30_ treatment; however, under the S_2_ gradient, no significant difference was observed between the N_30_ and N_45_ treatments. Compared with S_0_N_0_, the S_2_N_45_ treatment significantly increased plant height by 326.8%, stem diameter by 152.0%, and root length by 334.8%. Compared with S_2_N_0_, the plant height, stem diameter, and root length under the S_2_N_45_ treatment were significantly increased by 214.2%, 61.7%, and 179.7%, respectively.

**Figure 2:**
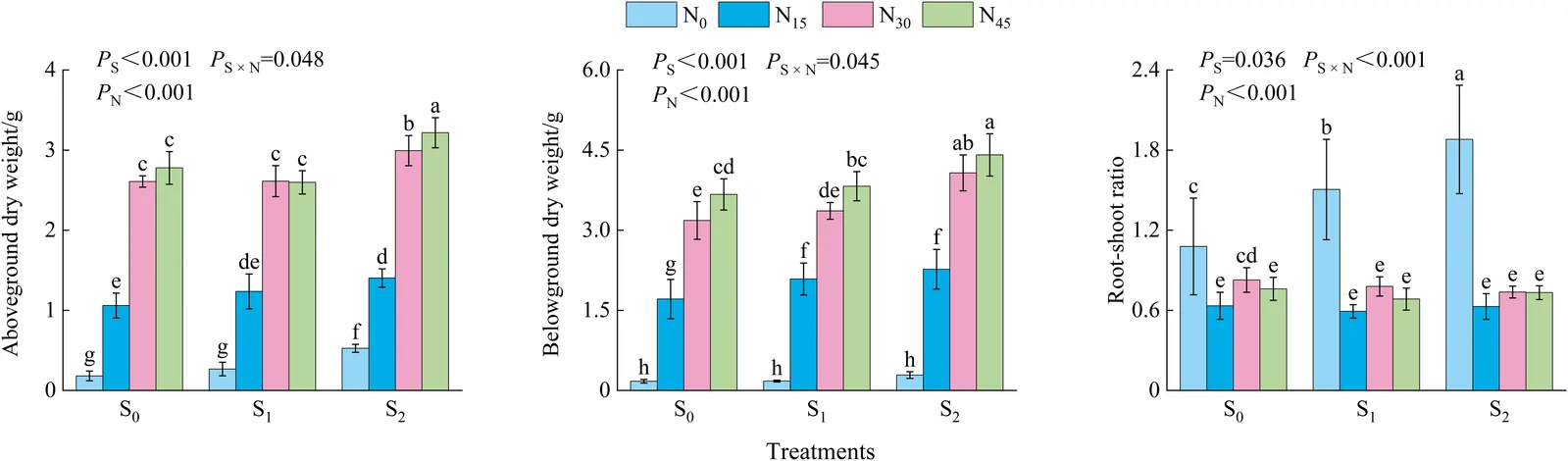
Effects of soil salinity and nitrogen addition on biomass and root-to-shoot ratio of *L. bicolor*. S_0_–S_2_ represent soil salt contents of blank control, 0.3%, and 0.6%, respectively; N_0_–N_45_ represent nitrogen addition levels of 0, 15, 30, and 45 kg·N·ha^−1^·yr^−1^, respectively; S denotes salt gradient, N denotes nitrogen addition level, and S×N denotes the combined application of salt and nitrogen; different lowercase letters indicate significant differences among treatments (*P* < 0.05).

Different salt and nitrogen treatments had significant effects on the biomass per plant of *L. bicolor*, and the biomass response to salt–nitrogen coupling was more sensitive (**Figure 2**). Compared with the S_0_N_0_ treatment, the S_2_N_45_ treatment significantly increased aboveground dry weight from 0.17 g·plant^−1^ to 3.22 g·plant^−1^, and belowground dry weight from 0.17 g·plant^−1^ to 4.41 g·plant^−1^. Under the same salinity level, the aboveground and belowground dry weights in the S_2_N_45_ treatment were significantly higher than those in the S_2_N_0_ treatment by 511.9% and 1427.4%, respectively; however, no significant differences were observed between the S_1_N_30_ and S_1_N_45_ treatments, nor between the S_0_N_30_ and S_0_N_45_ treatments. At the same nitrogen addition level, the aboveground and belowground dry weights in the S_2_N_45_ treatment were significantly higher than those in the S_1_N_45_ treatment by 23.8% and 15.3%, respectively, whereas no significant differences were found between the S_0_N_45_ and S_1_N_45_ treatments, or between the S_0_N_30_ and S_1_N_30_ treatments. The root-to-shoot ratio increased significantly with increasing salinity under the N_0_ treatment, but showed no significant differences among salinity levels under the N_15_ to N_45_ treatments.

### 3.2 Effects of salt–nitrogen on photosynthetic characteristics of *L. bicolor*

#### 3.2.1 Gas exchange parameters

**Figure 3:**
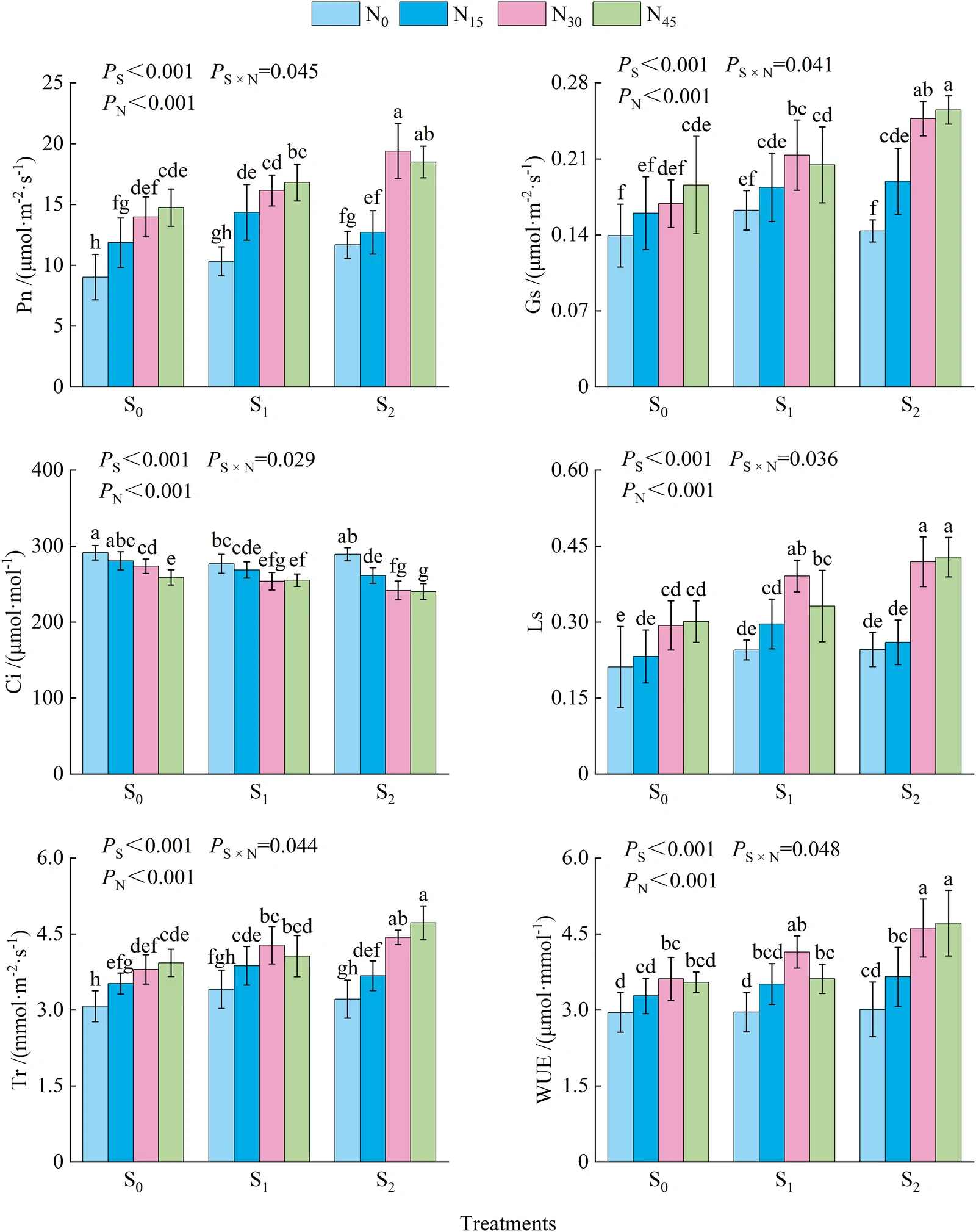
Effects of soil salinity and nitrogen addition on leaf photosynthetic characteristics of *L. bicolor*. S_0_–S_2_ represent soil salt contents of blank control, 0.3%, and 0.6%, respectively; N_0_–N_45_ represent nitrogen addition levels of 0, 15, 30, and 45 kg·N·ha^−1^·yr^−1^, respectively; S denotes salt gradient, N denotes nitrogen addition level, and S×N denotes the combined application of salt and nitrogen; different lowercase letters indicate significant differences among treatments (*P* < 0.05).

Salt, nitrogen, and their interaction had highly significant effects on the *P*_n_ of *L. bicolor* leaves (**Figure 3**). The variation in *P*_n_ exhibited a high degree of consistency with plant height and biomass. The *P*_n_ reached the highest levels under the S_2_N_30_ and S_2_N_45_ treatments, with no significant difference between these two treatments, and was significantly increased by 114.8% and 104.9%, respectively, compared with the S_0_N_0_ treatment. Under the S_2_ condition, the *P*_n_ in the N_30_ treatment was significantly increased by 65.8% compared with N_0_; under the N_30_ condition, the *P*_n_ in the S_2_ treatment was significantly increased by 38.7% compared with S_0_.

Different salinity-nitrogen treatments had significant effects on *G*_s_, *C*_i_, and *L*_s_ of *L. bicolor*, and there was a significant interaction between salinity and nitrogen addition (**Figure 3**). Under the same nitrogen addition level, *G*_s_ in the S_2_ treatment was significantly higher than that in the S_0_ and S_1_ treatments; *G*_s_ in the S_2_N_45_ treatment was significantly increased by 83.0% compared with the S_0_N_0_ treatment. No significant difference was observed between the S_2_N_45_ and S_2_N_30_ treatments, but significant differences were found between S_2_N_45_ and all other treatments. In contrast to the changes in *G*_s_, *C*_i_ in the S_2_N_45_ treatment decreased by 21.3% relative to the S_0_N_0_ treatment. *L*_s_ was significantly negatively correlated with *C*_i_, and *L*_s_ in the S_2_N_45_ treatment increased by 102.7% compared with the S_0_N_0_ treatment.

Different salinity-nitrogen treatments also had significant effects on *T*_r_ and WUE, with a significant interaction between salinity and nitrogen addition (**Figure 3**). The variation trend of *T*_r_ was generally consistent with that of *G*_s_; *T*_r_ in the S_2_N_45_ treatment was significantly increased by 53.6% compared with the S_0_N_0_ treatment, and was significantly higher by 16.2% and 20.1% than in the S_1_N_45_ and S_0_N_45_ treatments, respectively, and by 46.8% and 28.4% compared with the S_2_N_0_ and S_2_N_15_ treatments, respectively. However, no significant difference was found between the S_2_N_45_ and S_2_N_30_ treatments. Under the S_2_N_45_ treatment, WUE was significantly increased by 70.6%, 33.0%, and 56.5% compared with the S_0_N_0_, S_0_N_45_, and S_2_N_0_ treatments, respectively, while no significant difference was observed between S_2_N_45_ and S_2_N_30_.

#### 3.2.2 SPAD value and photosynthetic pigment contents in leaves of *L. bicolor*

Different salt and nitrogen treatments, as well as their interaction, significantly regulated the SPAD value of *L. bicolor* leaves (**Table 1**). The SPAD value in the S_2_N_45_ treatment was significantly increased by 47.6% compared with the S_0_N_0_ treatment. Under the S_0_ and S_1_ salinity treatments, the SPAD values followed the order of N_0_<N_15_<N_30_, while no significant difference was observed between N_30_ and N_45_. Compared with the S_2_N_30_, S_2_N_15_, and S_2_N_0_ treatments, the SPAD value under the S_2_N_45_ treatment was significantly increased by 5.1%–35.6%; compared with the S_1_N_45_ and S_0_N_45_ treatments, the SPAD value under the S_2_N_45_ treatment was significantly increased by 3.6% and 14.6%, respectively.

**Table 1:** Effects of soil salinity and nitrogen addition on SPAD values and photosynthetic pigment contents in leaves of *L. bicolor*.

Treatments	SPAD	Chlorophyll a/ (mg·g^-1^)	Chlorophyll b/ (mg·g^-1^)	Carotenoids/ (mg·g^-1^)	Chlorophyll a+b/ (mg·g^-1^)
S_0_N_0_	43.75 ± 1.36 g	1.50 ± 0.16 e	0.26 ± 0.06 e	0.68 ± 0.13 e	1.76 ± 0.12 g
S_0_N_15_	51.28 ± 1.27 e	1.74 ± 0.64 de	0.29 ± 0.08 e	0.75 ± 0.21 de	2.04 ± 0.68 fg
S_0_N_30_	57.33 ± 1.43 c	1.73 ± 0.36 de	0.33 ± 0.08 de	1.00 ± 0.31 cde	2.06 ± 0.39 efg
S_0_N_45_	56.35 ± 1.18 c	2.26 ± 0.38 cd	0.52 ± 0.10 b	1.11 ± 0.21 cd	2.79 ± 0.41 cde
S_1_N_0_	46.46 ± 1.43 f	1.91 ± 0.44 de	0.33 ± 0.04 de	0.70 ± 0.16 e	2.24 ± 0.46 defg
S_1_N_15_	54.11 ± 2.85 d	2.34 ± 0.37 cd	0.36 ± 0.09 de	1.00 ± 0.16 cde	2.70 ± 0.34 cdef
S_1_N_30_	61.81 ± 0.60 b	2.83 ± 0.59 bc	0.41 ± 0.07 cd	1.52 ± 0.23 ab	3.24 ± 0.55 bc
S_1_N_45_	62.35 ± 1.29 b	2.43 ± 0.68 cd	0.48 ± 0.08 bc	1.50 ± 0.18 ab	2.90 ± 0.63 cd
S_2_N_0_	47.61 ± 1.52 f	2.01 ± 0.26 de	0.35 ± 0.07 de	1.20 ± 0.62 bc	2.36 ± 0.25 defg
S_2_N_15_	57.60 ± 1.67 c	3.38 ± 0.63 ab	0.40 ± 0.07 cd	1.61 ± 0.30 a	3.78 ± 0.62 ab
S_2_N_30_	61.46 ± 0.79 b	3.73 ± 0.66 a	0.52 ± 0.06 b	1.71 ± 0.27 a	4.25 ± 0.65 a
S_2_N_45_	64.58 ± 1.76 a	3.24 ± 0.72 ab	0.66 ± 0.10 a	1.34 ± 0.22abc	3.90 ± 0.73 ab
S	***	***	***	***	***
N	***	***	***	***	***
S×N	**	*	NS	*	*

S_0_–S_2_ represent soil salt contents of blank control, 0.3%, and 0.6%, respectively; N_0_–N_45_ represent nitrogen addition levels of 0, 15, 30, and 45 kg·N·ha^−1^·yr^−1^, respectively; S denotes salt gradient, N denotes nitrogen addition level, and S×N denotes the combined application of salt and nitrogen; ***P ≤ 0.001, **P ≤ 0.01, *P ≤ 0.05, NS indicates no significant difference.

Different salt and nitrogen additions had highly significant effects on the Chl a, Chl b, Car, and Chl a+b contents of *L. bicolor*, and significant interactive effects of salt and nitrogen were observed for Chl a, Car, and Chl a+b (**Table 1**). Under the S_2_N_30_ treatment, the Chl a content increased by 85.6% compared with S_2_N_0_, and by 115.6% compared with S_0_N_30_. For Chl b content, the S_2_N_45_ treatment increased Chl b content by 88.6% and 26.9% compared with the S_2_N_0_ and S_0_N_45_ treatments, respectively. Compared with S_0_N_45_, the Chl a+b content under S_2_N_45_ increased by 39.8%; the Chl a+b under the S_2_N_45_ and S_2_N_30_ treatments was significantly higher than that under S_2_N_0_, with increases of 65.3% and 80.1%, respectively. The carotenoid content under the S_2_N_30_ treatment was not significantly different from that under the S_2_N_15_ treatment, but both were significantly higher than that under the S_2_N_0_ treatment, with increases of 42.5% and 34.2% for S_2_N_30_ and S_2_N_15_, respectively, relative to S_2_N_0_.

### 3.3 Effects of salt–nitrogen interaction on element contents and ecological stoichiometric characteristics of *L. bicolor*

**Figure 4:**
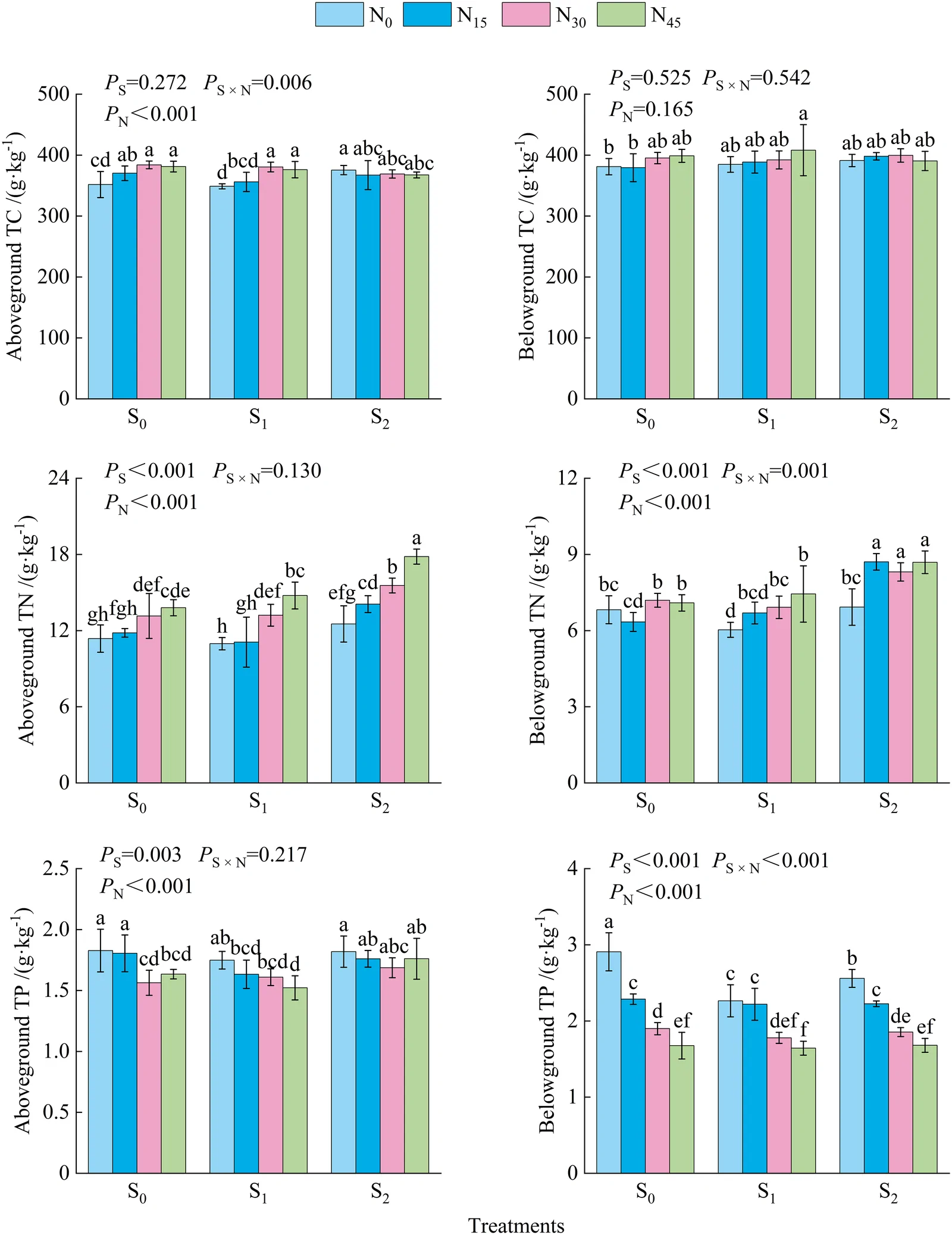
Effects of soil salinity and nitrogen addition on TC, TN, and TP contents of *L. bicolor*. S_0_–S_2_ represent soil salt contents of blank control, 0.3%, and 0.6%, respectively; N_0_–N_45_ represent nitrogen addition levels of 0, 15, 30, and 45 kg·N·ha^−1^·yr^−1^, respectively; S denotes salt gradient, N denotes nitrogen addition level, and S×N denotes the combined application of salt and nitrogen; different lowercase letters indicate significant differences among treatments (*P* < 0.05).

As shown in **Figure 4**, the aboveground TC content remained relatively stable, with a significant salt–nitrogen interaction effect but no significant main effect of salinity. The aboveground TN content was highly significantly regulated by the salt–nitrogen interaction; the aboveground TN content in the S_2_N_45_ treatment was significantly higher than that in the other treatments by 14.7%–62.6%. The aboveground TP content was highly significantly regulated by both salinity and nitrogen, showing an overall decreasing trend with nitrogen addition; compared with the S_0_N_0_ treatment, the S_0_N_45_ treatment exhibited a significant decrease of 11.8%, and compared with the S_1_N_15_ treatment, the S_0_N_15_ treatment showed a significant decrease of 17.2%.

No significant differences in belowground TC content were observed among treatments. The belowground TN content was highly significantly regulated by the salt–nitrogen interaction; the belowground TN contents in the S_2_N_15_, S_2_N_30_, and S_2_N_45_ treatments were significantly increased, with no significant differences among these three treatments. However, under the S_2_ treatment, the belowground TN contents under nitrogen addition treatments were significantly higher than those under the S_0_ and S_1_ treatments. The belowground TP content was highly significantly affected by the salt–nitrogen interaction, showing a significant decreasing trend overall with nitrogen addition; the belowground TP content in the S_1_N_45_ treatment was significantly reduced by 77.1% compared with the S_0_N_0_ treatment.

**Figure 5:**
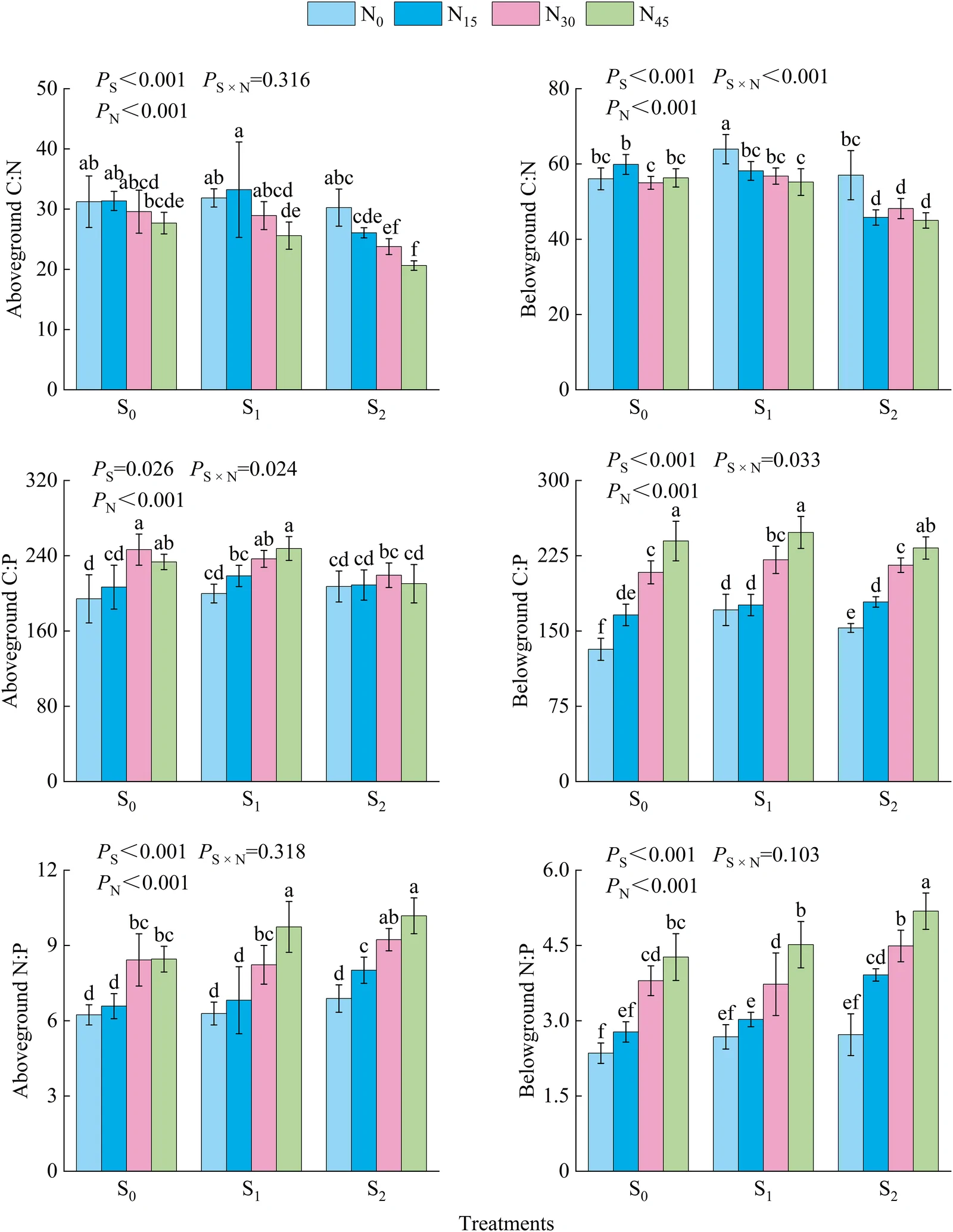
Effects of soil salinity and nitrogen addition on ecological stoichiometric characteristics of *L. bicolor*. S_0_–S_2_ represent soil salt contents of blank control, 0.3%, and 0.6%, respectively; N_0_–N_45_ represent nitrogen addition levels of 0, 15, 30, and 45 kg·N·ha^−1^·yr^−1^, respectively; S denotes salt gradient, N denotes nitrogen addition level, and S×N denotes the combined application of salt and nitrogen; different lowercase letters indicate significant differences among treatments (*P* < 0.05).

As shown in **Figure 5**, the aboveground C:N ratio exhibited a significant decreasing trend with increasing nitrogen addition, with the ratio in the S_2_N_45_ treatment being significantly decreased by 61.1% compared with the S_1_N_15_ treatment. The aboveground C:P ratio was significantly regulated by the salt–nitrogen interaction, and was significantly increased by 26.6%–27.5% in the S_0_N_30_ and S_1_N_45_ treatments relative to the S_0_N_0_ treatment. The aboveground N:P ratio showed a significant increasing trend with increasing salinity and nitrogen addition; the ratios in the S_2_N_45_ and S_1_N_45_ treatments were significantly increased by 63.3% and 56.2%, respectively, compared with the S_0_N_0_ treatment. For the belowground parts, the C:N ratio had a significant response to the salt–nitrogen interaction; under the S_2_ treatment, the C:N ratios at N_15_–N_45_ were significantly decreased, and the belowground C:N ratio in the S_2_N_45_ treatment was significantly reduced by 42.1% compared with the S_1_N_0_ treatment. The belowground C:P ratio showed an increasing trend with increasing nitrogen addition, with a significant salt–nitrogen interaction; it was significantly increased by 79.2%–85.6% in the S_0_N_45_ and S_1_N_45_ treatments relative to the S_0_N_0_ treatment. The belowground N:P ratio exhibited an increasing trend with increasing salinity and nitrogen addition; the belowground N:P ratio in the S_2_N_45_ treatment was significantly increased by 14.8%–120.3% compared with the other treatments.

**Figure 6:**
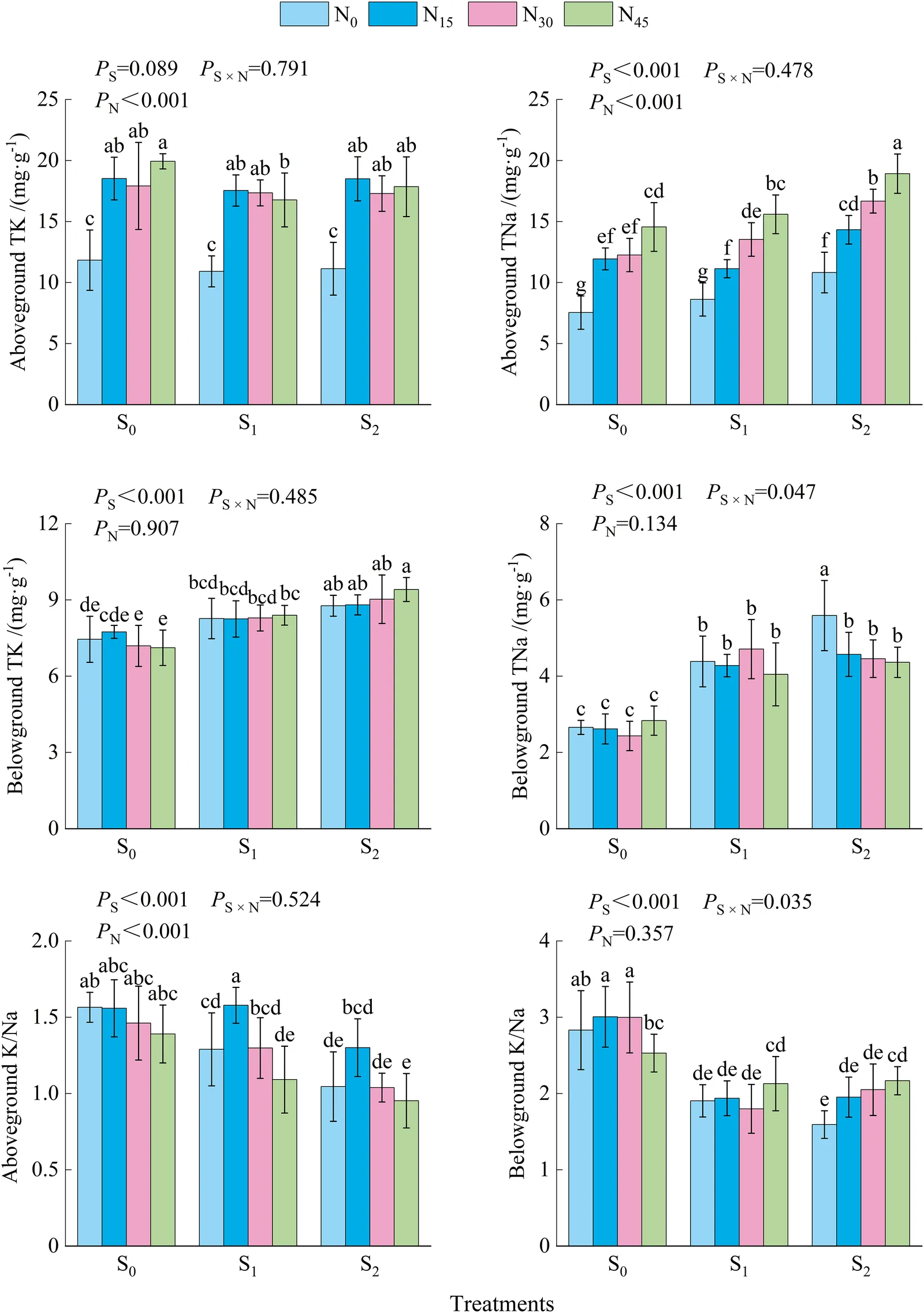
Effects of soil salinity and nitrogen addition on TK, TNa contents and the K/Na ratio of *L. bicolor*. S_0_–S_2_ represent soil salt contents of blank control, 0.3%, and 0.6%, respectively; N_0_–N_45_ represent nitrogen addition levels of 0, 15, 30, and 45 kg·N·ha^−1^·yr^−1^, respectively; S denotes salt gradient, N denotes nitrogen addition level, and S×N denotes the combined application of salt and nitrogen; different lowercase letters indicate significant differences among treatments (*P* < 0.05).

As shown in **Figure 6**, the interaction between salt and nitrogen significantly affected the absorption and distribution of potassium and sodium ions in *L. bicolor*. Aboveground potassium content was highly significantly regulated by the main effect of nitrogen; regardless of salinity level, nitrogen application significantly increased aboveground potassium accumulation, with the potassium content under the S_0_N_45_ treatment being significantly increased by 68.4% compared with S_0_N_0_. Aboveground sodium content was highly significantly affected by both salinity and nitrogen addition, showing an increasing trend with rising salinity and nitrogen addition levels. Specifically, the S_2_N_45_ treatment significantly increased by 151.0% and 74.9% compared with the S_0_N_0_ and S_2_N_0_ treatments, respectively. Belowground potassium content responded significantly only to salinity; the S_2_N_45_ treatment increased by 32.2% and 12.1% compared with the S_0_N_45_ and S_1_N_45_ treatments, respectively. Belowground sodium content was highly significantly influenced by the main effect of salinity, with a significant salt–nitrogen interaction; under S_2_ conditions (N_15_–N_45_ treatments), the sodium content was significantly lower than that in the S_2_N_0_ treatment. The aboveground K/Na ratio was highly significantly affected by salinity and nitrogen, with the S_2_N_45_ treatment decreasing by 64.3% compared with the S_0_N_0_ treatment. Salinity had a highly significant effect on the belowground K/Na ratio, and the salt–nitrogen interaction was also significant. Specifically, the K/Na ratios in the S_0_N_15_ and S_0_N_30_ treatments were significantly higher than those in the S_2_N_15_ and S_2_N_30_ treatments, whereas the ratio in the S_2_N_45_ treatment was significantly higher than that in the S_2_N_0_ treatment.

### 3.4 Effects of salt–nitrogen addition on soil physicochemical properties

#### 3.4.1 Soil salinization characteristics and pH

**Figure 7:**
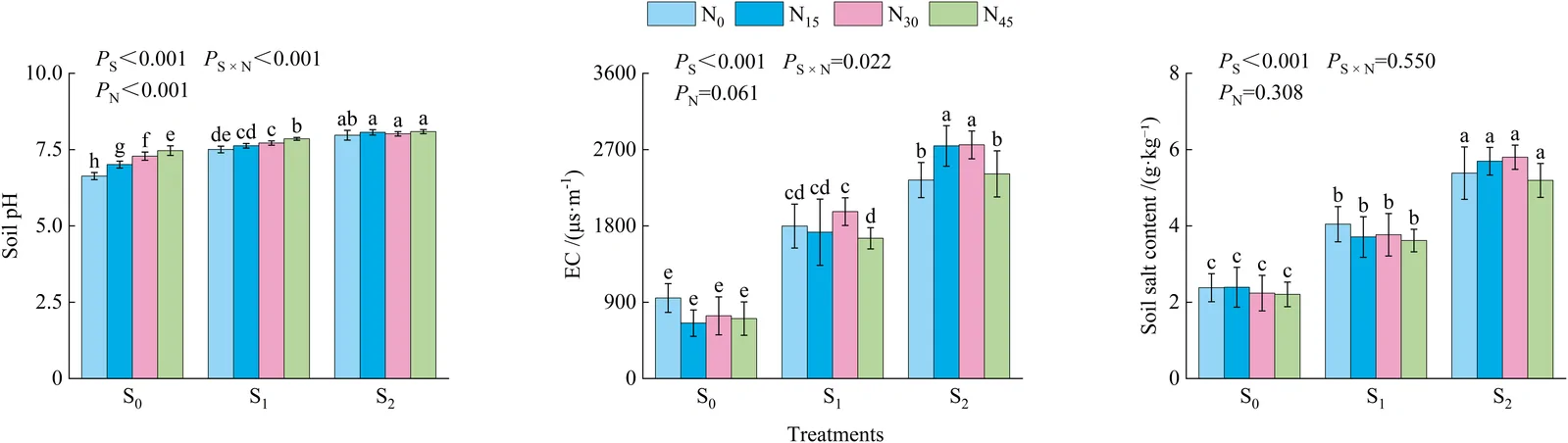
Effects of soil salinity and nitrogen addition on soil pH, EC, and salt content. S_0_–S_2_ represent soil salt contents of blank control, 0.3%, and 0.6%, respectively; N_0_–N_45_ represent nitrogen addition levels of 0, 15, 30, and 45 kg·N·ha^−1^·yr^−1^, respectively; S denotes salt gradient, N denotes nitrogen addition level, and S×N denotes the combined application of salt and nitrogen; different lowercase letters indicate significant differences among treatments (*P* < 0.05).

Salinity addition exerted highly significant promoting effects on soil pH, EC, and total water-soluble salt content of *L. bicolor*; nitrogen addition alone showed a significant effect only on soil pH, while the salt–nitrogen interaction significantly regulated soil pH and EC (**Figure 7**). Soil pH exhibited an increasing trend with rising salinity gradients; under the N_0_ condition, pH in the S_2_ treatment was significantly increased by 20.2% compared with the S_0_ treatment. Under the S_0_ salinity level, soil pH gradually increased as nitrogen addition rose from N_0_ to N_45_, with pH values in the N_45_ treatment being elevated by 12.6% and 4.6% under S_0_ and S_1_ conditions, respectively, relative to the N_0_ treatment.

The responses of soil EC and total water-soluble salt content to salt–nitrogen addition followed the same pattern, both showing a significant increasing trend with rising salinity gradients. Under the S_2_ and S_1_ treatments, EC and total water-soluble salt content were elevated compared with the S_0_ treatment. Specifically, EC in the S_2_N_15_ and S_2_N_30_ treatments were increased by 17.2% and 17.7%, respectively, relative to the S_2_N_0_ treatment, and by 13.7% and 14.2%, respectively, compared with the S_2_N_45_ treatment, exhibiting a trend of first increasing and then decreasing. However, under each salinity gradient, different nitrogen addition treatments had no significant effect on soil total water-soluble salt content.

#### 3.4.2 Soil available nutrients

**Figure 8:**
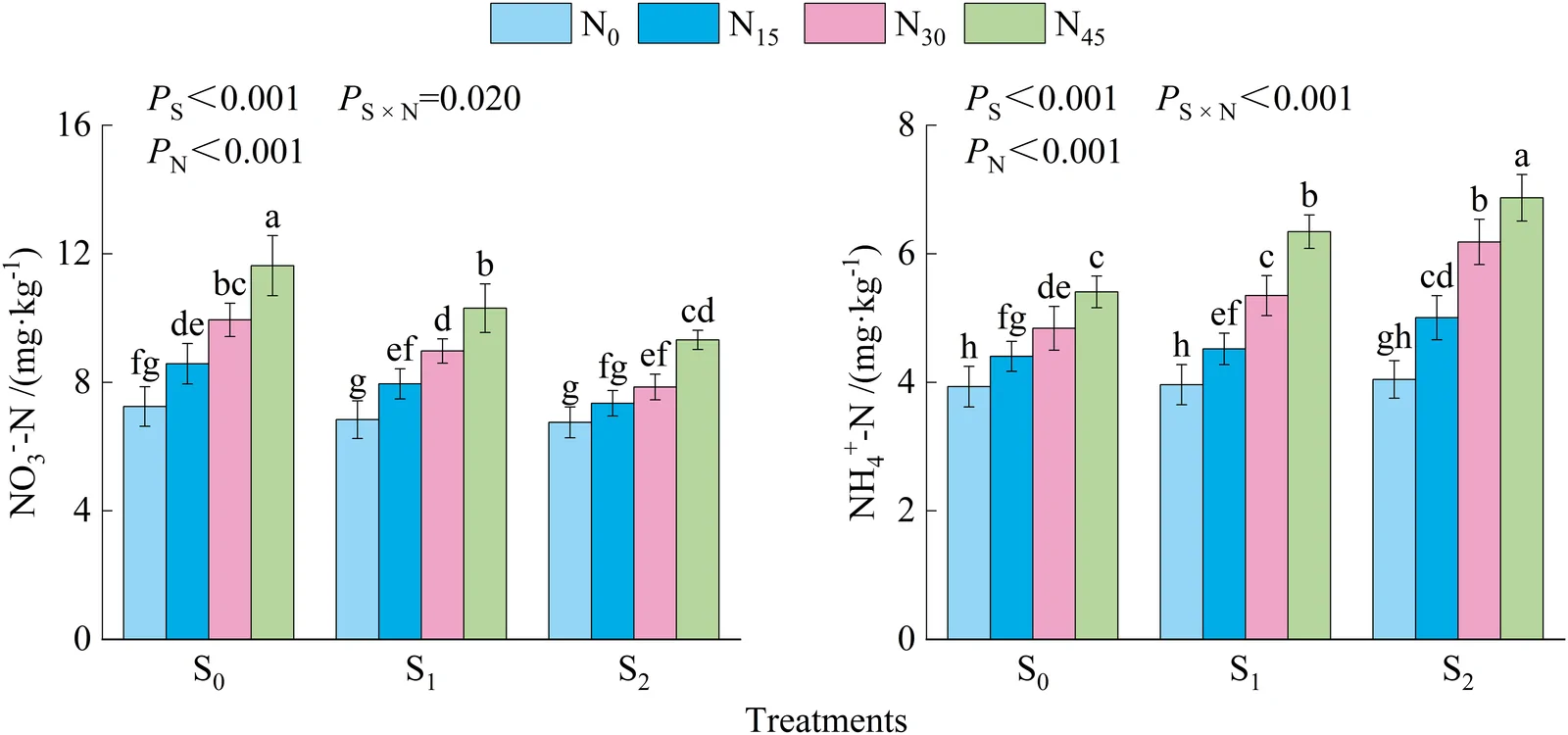
Effects of soil salinity and nitrogen addition on soil inorganic nitrogen content. S_0_–S_2_ represent soil salt contents of blank control, 0.3%, and 0.6%, respectively; N_0_–N_45_ represent nitrogen addition levels of 0, 15, 30, and 45 kg·N·ha^−1^·yr^−1^, respectively; S denotes salt gradient, N denotes nitrogen addition level, and S×N denotes the combined application of salt and nitrogen; different lowercase letters indicate significant differences among treatments (*P* < 0.05).

Salinity, nitrogen addition, and their interaction all significantly affected soil NH_4_^+^-N and NO_3_^−^-N contents (**Figure 8**). Both salinity and nitrogen exhibited highly significant main effects on NH_4_^+^-N, with a significant interaction between the two factors. Under the same salinity level, NH_4_^+^-N content showed an increasing trend with rising nitrogen addition, following the order N_0_< N_15_< N_30_< N_45_. Under the S_0_ and S_2_ conditions, the NH_4_^+^-N content in the N_45_ treatment was significantly increased by 37.5% and 69.9%, respectively, compared with the N_0_ treatment. Salinity and nitrogen also significantly affected NO_3_^−^-N content, with a significant interaction. Unlike NH_4_^+^-N, increased salinity exhibited a clear inhibitory effect on NO_3_^−^-N accumulation. Under the N_45_ condition, NH_4_^+^-N content under the S_0_ treatment was 14.8% and 21.3% lower than that under the S1 and S2 treatments, respectively. Under the S_0_ condition, the NO_3_^−^-N content exhibited a relatively large increase with rising nitrogen addition, with the N_45_ treatment showing a significant increase of 60.5% compared with N_0_; however, under the S_2_ condition, the NO_3_^−^-N content in the N_45_ treatment increased by only 38.0% relative to N_0_, representing a markedly reduced magnitude of increase.

**Figure 9:**
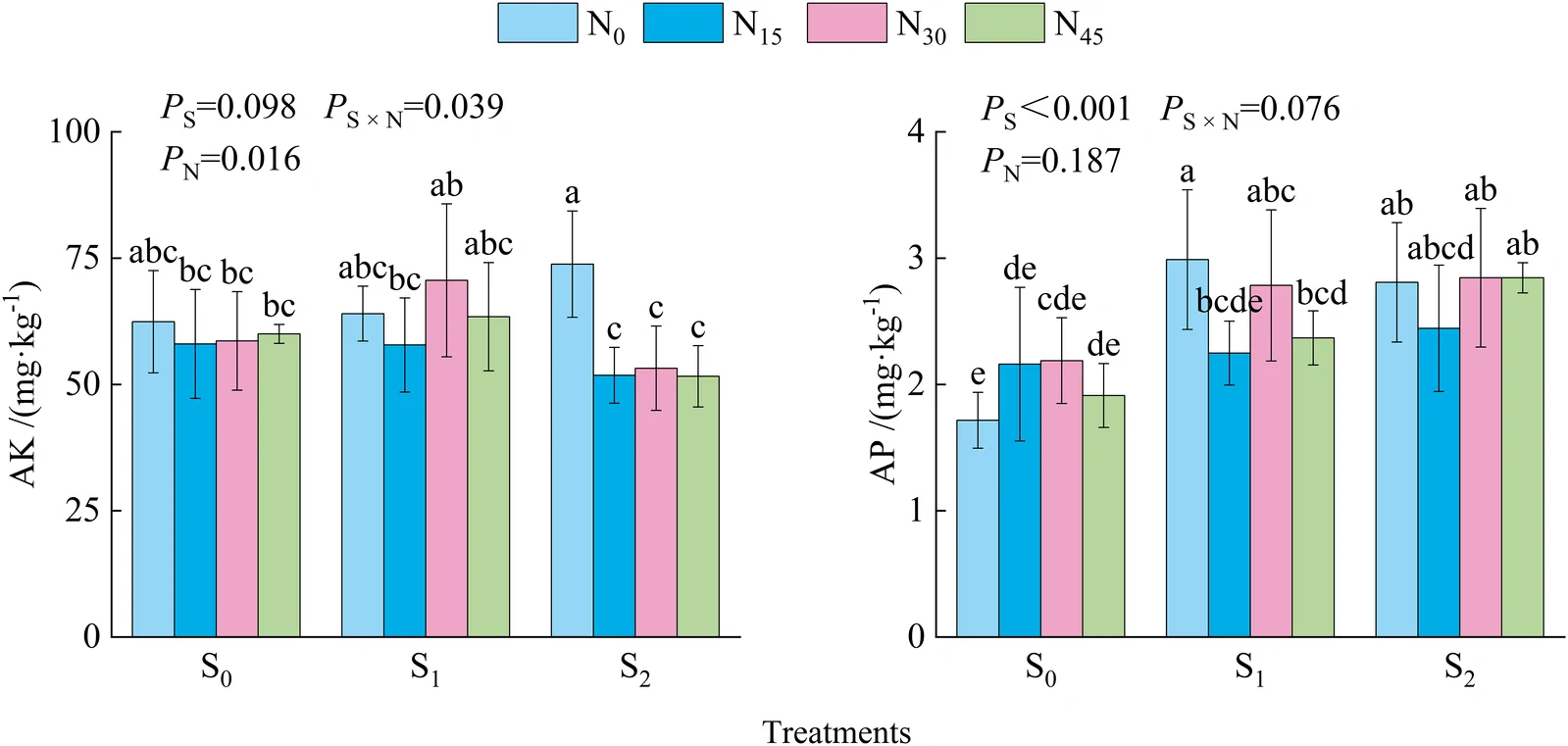
Effects of soil salinity and nitrogen addition on soil AK and AP. S_0_–S_2_ represent soil salt contents of blank control, 0.3%, and 0.6%, respectively; N_0_–N_45_ represent nitrogen addition levels of 0, 15, 30, and 45 kg·N·ha^−1^·yr^−1^, respectively; S denotes salt gradient, N denotes nitrogen addition level, and S×N denotes the combined application of salt and nitrogen; different lowercase letters indicate significant differences among treatments (*P* < 0.05).

As shown in **Figure 9**, the response of soil available potassium to salt–nitrogen addition exhibited a pattern of increase under low salinity and decrease under moderate salinity. Specifically, the available potassium content in the S_2_N_0_ treatment was 43.0% higher than that in the S_2_N_45_ treatment, while no significant differences were observed among different nitrogen addition treatments under the S_0_ and S_1_ conditions. Soil AP showed a highly significant positive response to salinity addition; the AP content in the S_1_N_0_ treatment was significantly increased by 74.1% compared with the S_0_N_0_ treatment, whereas under the same salinity gradient, no significant differences were found among different nitrogen addition treatments.

#### 3.4.3 SOM, MBC and MBN contents

**Figure 10:**
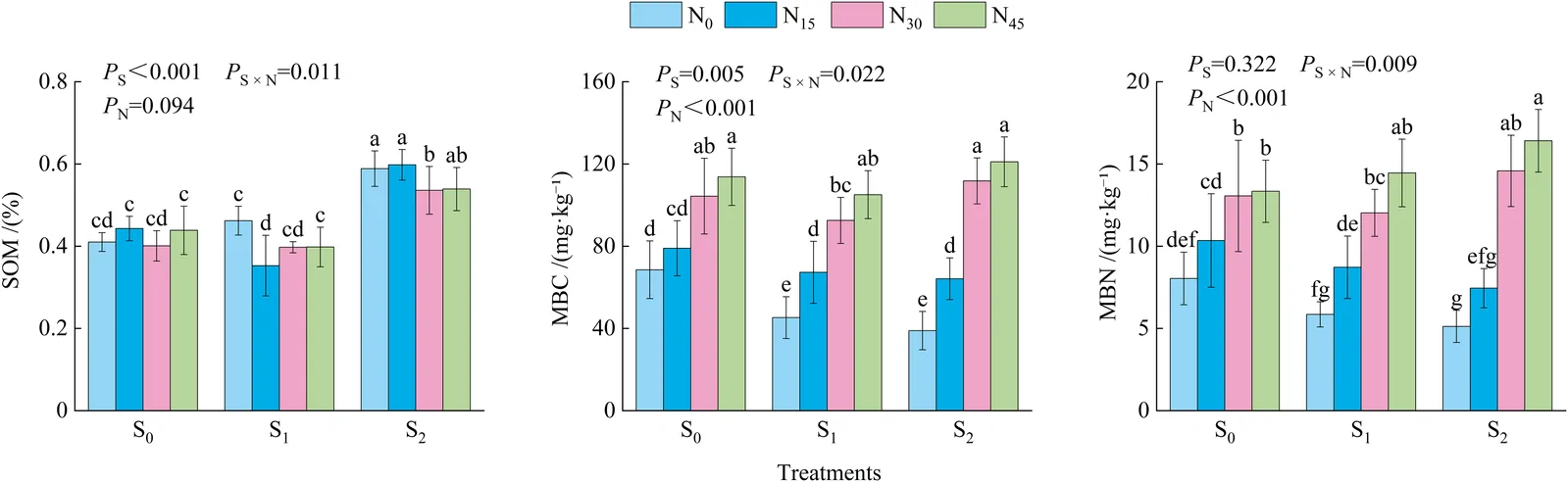
Effects of soil salinity and nitrogen addition on SOM, MBC, and MBN. S_0_–S_2_ represent soil salt contents of blank control, 0.3%, and 0.6%, respectively; N_0_–N_45_ represent nitrogen addition levels of 0, 15, 30, and 45 kg·N·ha^−1^·yr^−1^, respectively; S denotes salt gradient, N denotes nitrogen addition level, and S×N denotes the combined application of salt and nitrogen; different lowercase letters indicate significant differences among treatments (*P* < 0.05).

As shown in **Figure 10**, salt–nitrogen addition exerted significant regulatory effects on SOM, MBC, and MBN in the coastal saline soil of the Yellow River Delta. Salinity addition significantly promoted SOM and significantly affected MBC, but its effect on MBN did not reach a significant level. Nitrogen addition had highly significant promoting effects on MBC and MBN. The salt–nitrogen interaction exhibited significant regulatory effects on all three variables. SOM content showed an increasing trend with rising salinity gradients, with higher SOM content under the S_2_ treatment compared with S_0_ and S_1_. Under the S_0_N_45_, S_1_N_45_, and S_2_N_45_ treatments, MBC was increased by 66.0%, 132.2%, and 210.9%, respectively, compared with the corresponding N_0_ treatments under the same salinity gradients. Under the S_2_N_45_ treatment, MBN was significantly increased by 220.3% and 23.0% compared with the S_2_N_0_ and S_0_N_45_ treatments, respectively.

#### 3.4.4 Soil TC, TN, TP, and ecological stoichiometric characteristics

**Figure 11:**
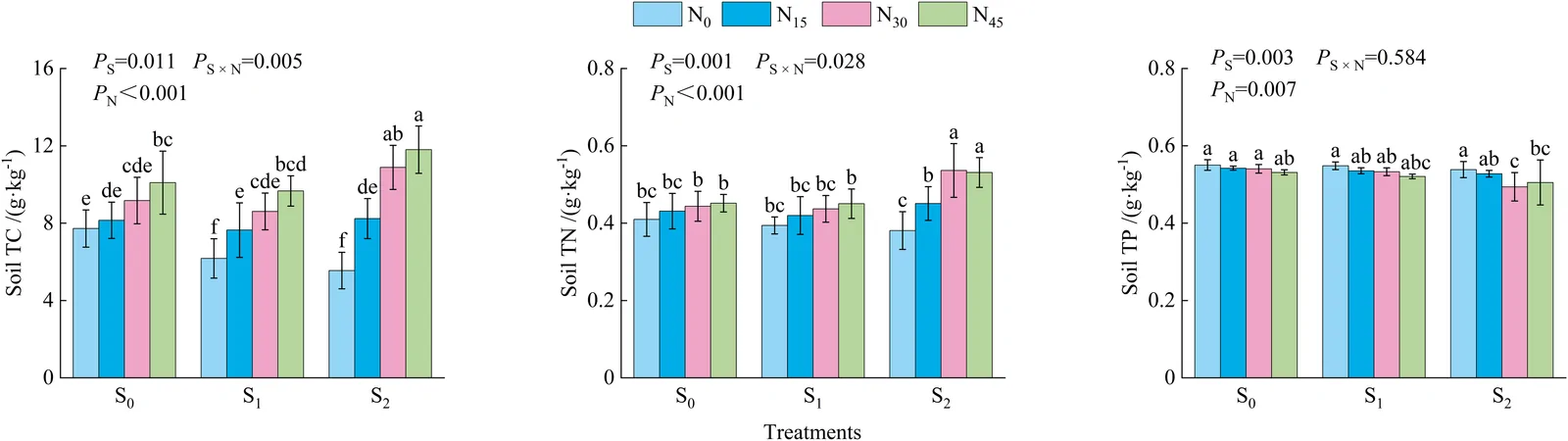
Effects of soil salinity and nitrogen addition on soil TC, TN, and TP contents. S_0_–S_2_ represent soil salt contents of blank control, 0.3%, and 0.6%, respectively; N_0_–N_45_ represent nitrogen addition levels of 0, 15, 30, and 45 kg·N·ha^−1^·yr^−1^, respectively; S denotes salt gradient, N denotes nitrogen addition level, and S×N denotes the combined application of salt and nitrogen; different lowercase letters indicate significant differences among treatments (*P* < 0.05).

As shown in **Figure 11**, different salt–nitrogen additions resulted in significant differences in soil TC, TN, and TP contents. Salinity addition had significant effects on all three variables. Nitrogen addition exhibited highly significant promoting effects on soil TC and TN, but showed a significant inhibitory effect on TP. The salt–nitrogen interaction exerted significant regulatory effects on TC and TN. Under the same salinity gradient, the S_0_N_45_, S_1_N_45_, and S_2_N_45_ treatments showed increases of 30.8%, 56.5%, and 112.8%, respectively, compared with the corresponding N_0_ treatments under the same salinity gradients. Under the S_2_N_30_ and S_2_N_45_ treatments, soil TN contents were increased by 40.8% and 39.4%, respectively, compared with the S_2_N_0_ treatment. Soil TP content remained relatively stable, with no significant differences observed among treatments except for the S_2_N_30_ treatment.

**Figure 12:**
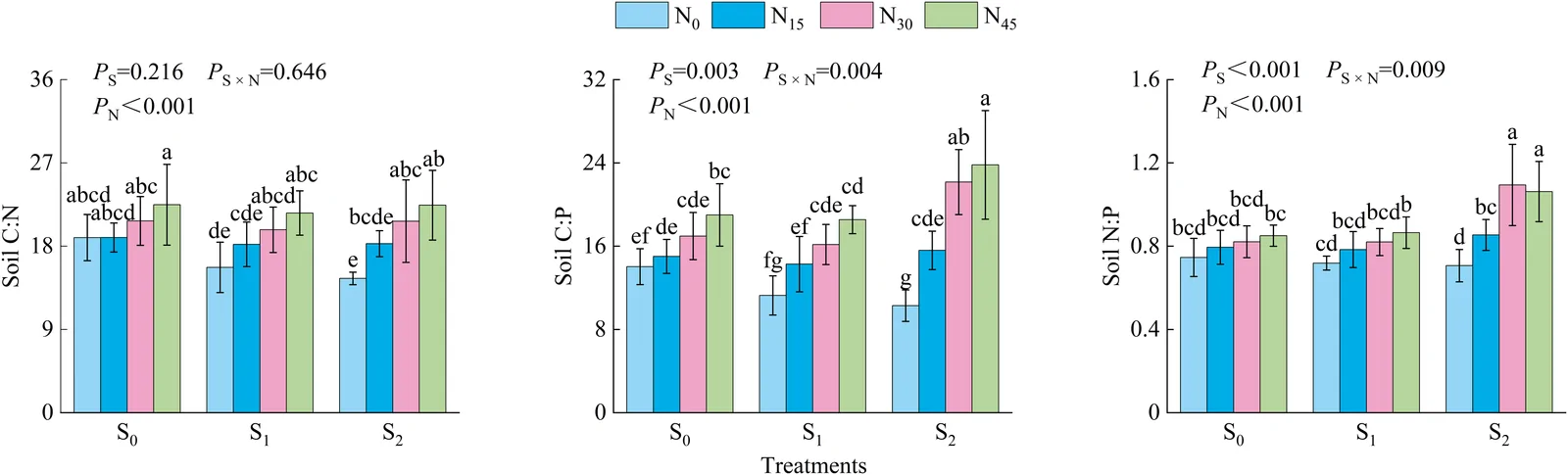
Effects of soil salinity and nitrogen addition on soil ecological stoichiometric characteristics. S_0_–S_2_ represent soil salt contents of blank control, 0.3%, and 0.6%, respectively; N_0_–N_45_ represent nitrogen addition levels of 0, 15, 30, and 45 kg·N·ha^−1^·yr^−1^, respectively; S denotes salt gradient, N denotes nitrogen addition level, and S×N denotes the combined application of salt and nitrogen; different lowercase letters indicate significant differences among treatments (*P* < 0.05).

As shown in **Figure 12**, salt–nitrogen addition exerted significant regulatory effects on soil ecological stoichiometric ratios. Salinity addition significantly affected C:P and exhibited a highly significant promoting effect on N:P. Nitrogen addition showed highly significant promoting effects on C:N, C:P, and N:P. The salt–nitrogen interaction significantly influenced C:P and N:P. For soil C:N, the S_0_N_45_ and S_2_N_45_ treatments were increased by 18.8% and 54.5%, respectively, compared with the corresponding N_0_ treatments under the same salinity gradients, with no significant salinity gradient differences observed among treatments. For soil C:P, the S_2_N_45_ treatment showed an increase of 131.6% compared with the S_2_N_0_ treatment. For soil N:P, the S_2_N_30_ and S_2_N_45_ treatments were increased by 54.9% and 50.4%, respectively, compared with the S_2_N_0_ treatment.

### 3.5 Correlation analysis of ecophysiological indicators of *L. bicolor*

**Figure 13:**
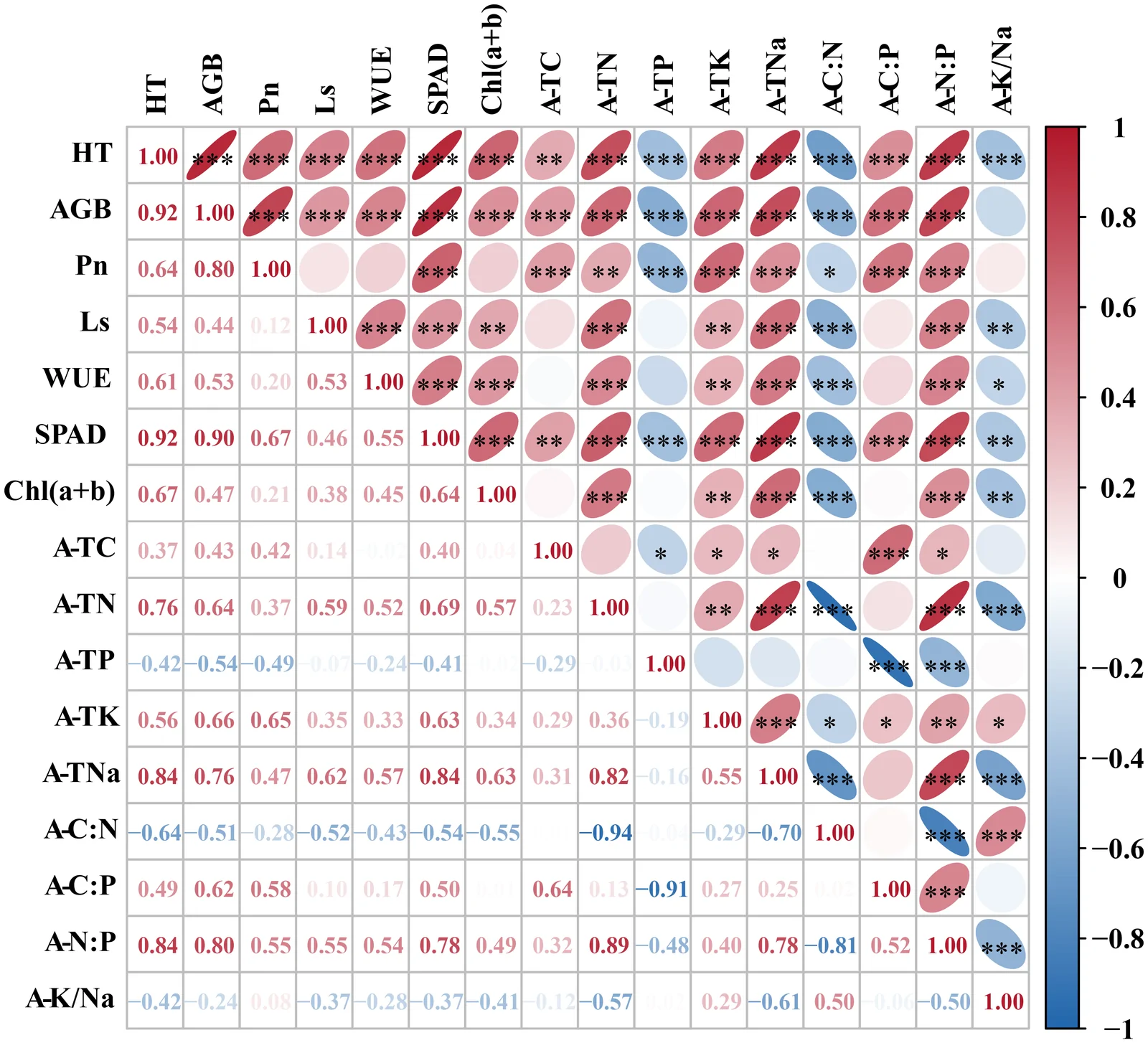
Correlation analysis of ecophysiological indicators of *L. bicolor* under different salinity and nitrogen addition treatments. The measured indicators included plant height (HT), aboveground biomass (AGB), net photosynthetic rate (*P*_n_), stomatal conductance (*G*_s_), water use efficiency (WUE), SPAD value, chlorophyll (a+b) content, aboveground carbon content (A-TC), aboveground nitrogen content (A-TN), aboveground phosphorus content (A-TP), aboveground potassium content (A-TK), aboveground sodium content (A-TNa), aboveground carbon-to-nitrogen ratio (A-C:N), aboveground carbon-to-phosphorus ratio (A-C:P), aboveground nitrogen-to-phosphorus ratio (A-N:P), and aboveground potassium-to-sodium ratio (A-K/Na). *P ≤ 0.05, **P ≤ 0.01, and ***P ≤ 0.001.

Pearson correlation analysis among the growth, photosynthetic, and elemental indices of *L. bicolor* under salt–nitrogen treatments revealed (**Figure 13**) that plant height (HT) was highly significantly positively correlated with aboveground biomass (AGB), and showed significant positive correlations with *P*_n_, *L*_s_, WUE, SPAD value, Chl(a+b), aboveground carbon content (A-TC), aboveground nitrogen content (A-TN), aboveground sodium content (A-TNa), aboveground potassium content (A-TK), and aboveground N:P ratio(A-N:P), whereas it was significantly negatively correlated with aboveground phosphorus content (A-TP), aboveground C:N ratio(A-C:N), and aboveground K/Na ratio(A-K/Na). *P*_n_ exhibited strong positive correlations with SPAD value, A-TC, A-TN, A-TNa, and A-TK, but negative correlations with A-TP and A-C:N. A-TN was highly significantly positively correlated with HT, *P*_n_, Chl(a+b), A-TNa, and A-TK, and highly significantly negatively correlated with A-C:N and A-K/Na. A-K/Na showed strong negative correlations with HT, *L*_s_, SPAD, Chl(a+b), A-TN, A-TNa, and A-N:P, and positive correlations with A-TK and A-C:N.

Under salt–nitrogen addition, the growth of *L. bicolor* was closely associated with photosynthetic capacity, chlorophyll content, and potassium/sodium accumulation. Specifically, HT and AGB were highly positively correlated with A-TN, negatively correlated with A-C:N, and positively correlated with A-N:P, indicating a synergistic adaptive pattern among carbon, nitrogen, and phosphorus elements, with nitrogen playing a dominant role.

### 3.6 Structural equation model of salt–nitrogen regulation on physiological and growth traits of *L. bicolor*

**Figure 14:**
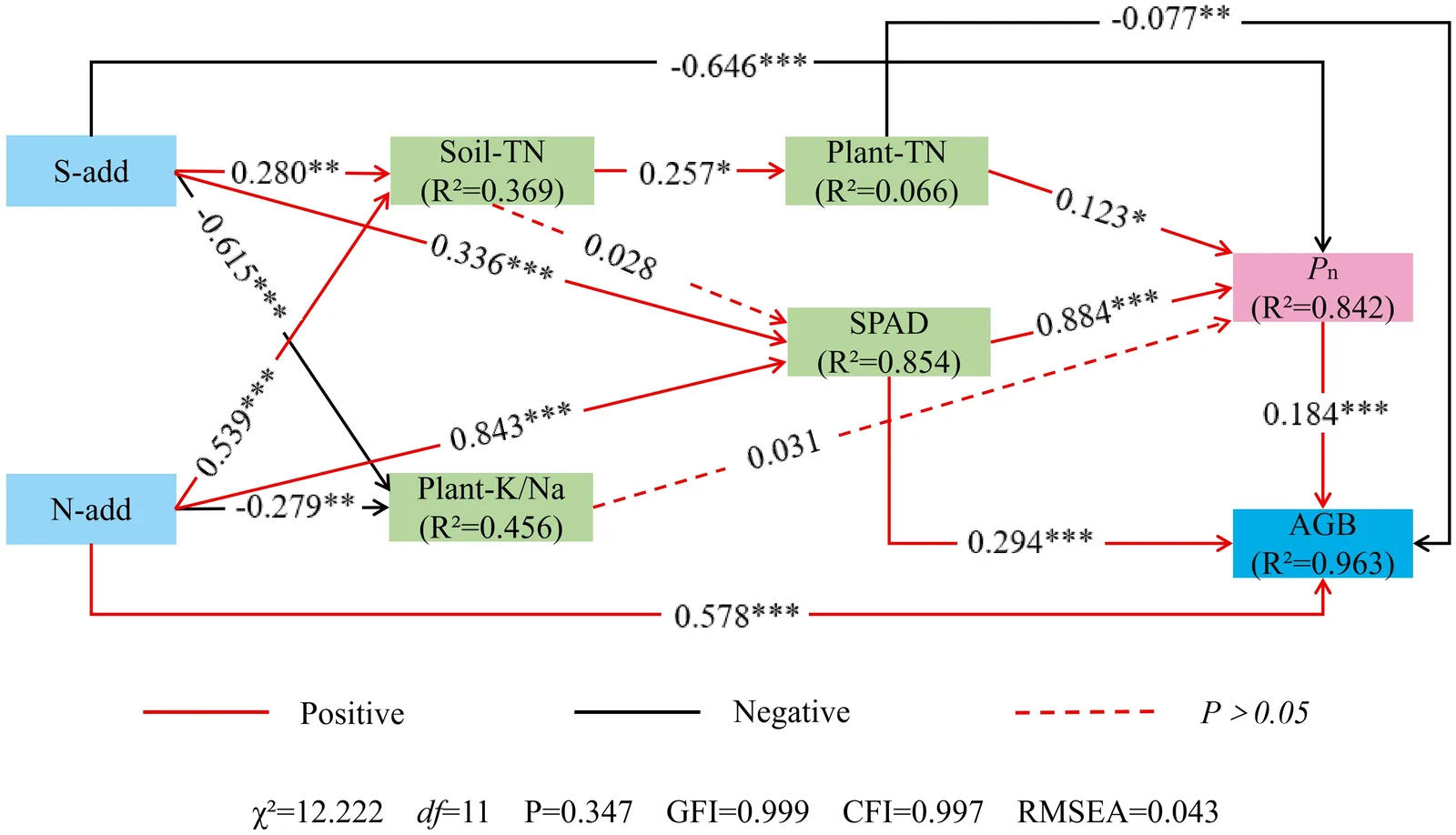
Structural equation model of the effects of soil salinity and nitrogen addition on physiological and growth traits of *L. bicolor*. salinity addition (S-add), nitrogen addition (N-add), soil total nitrogen (Soil-TN), aboveground total nitrogen (Plant-TN), aboveground K/Na ratio (Plant-K/Na), SPAD value, net photosynthetic rate (*P*_n_), and aboveground biomass (AGB). *P ≤ 0.05, **P ≤ 0.01, and ***P ≤ 0.001.

Based on the regulatory pathways of salt and nitrogen addition on the physiological and growth traits of *L. bicolor*, a structural equation model (SEM) was constructed (**Figure 14**). In the model, salt addition (S-add) and nitrogen addition (N-add) were set as exogenous variables; soil total nitrogen (Soil-TN), aboveground total nitrogen (Plant-TN), aboveground K/Na ratio (Plant-K/Na), SPAD value, and *P*_n_ were set as mediating variables; and aboveground biomass (AGB) was set as the final response variable. S-add had significant positive effects on Soil-TN and SPAD, and negative effects on Plant-K/Na and *P*_n_. N-add had significant positive effects on Soil-TN, SPAD, and AGB, and a negative effect on Plant-K/Na. Soil-TN had a positive effect on Plant-TN; Plant-TN had a positive effect on *P*_n_; SPAD had a significant direct effect on *P*_n_, and significant direct and indirect effects on AGB. S-add, N-add, SPAD, and *P*_n_ all had significant positive effects on the growth of *L. bicolor*, among which N-add had the largest positive effect.

**Figure 15:**
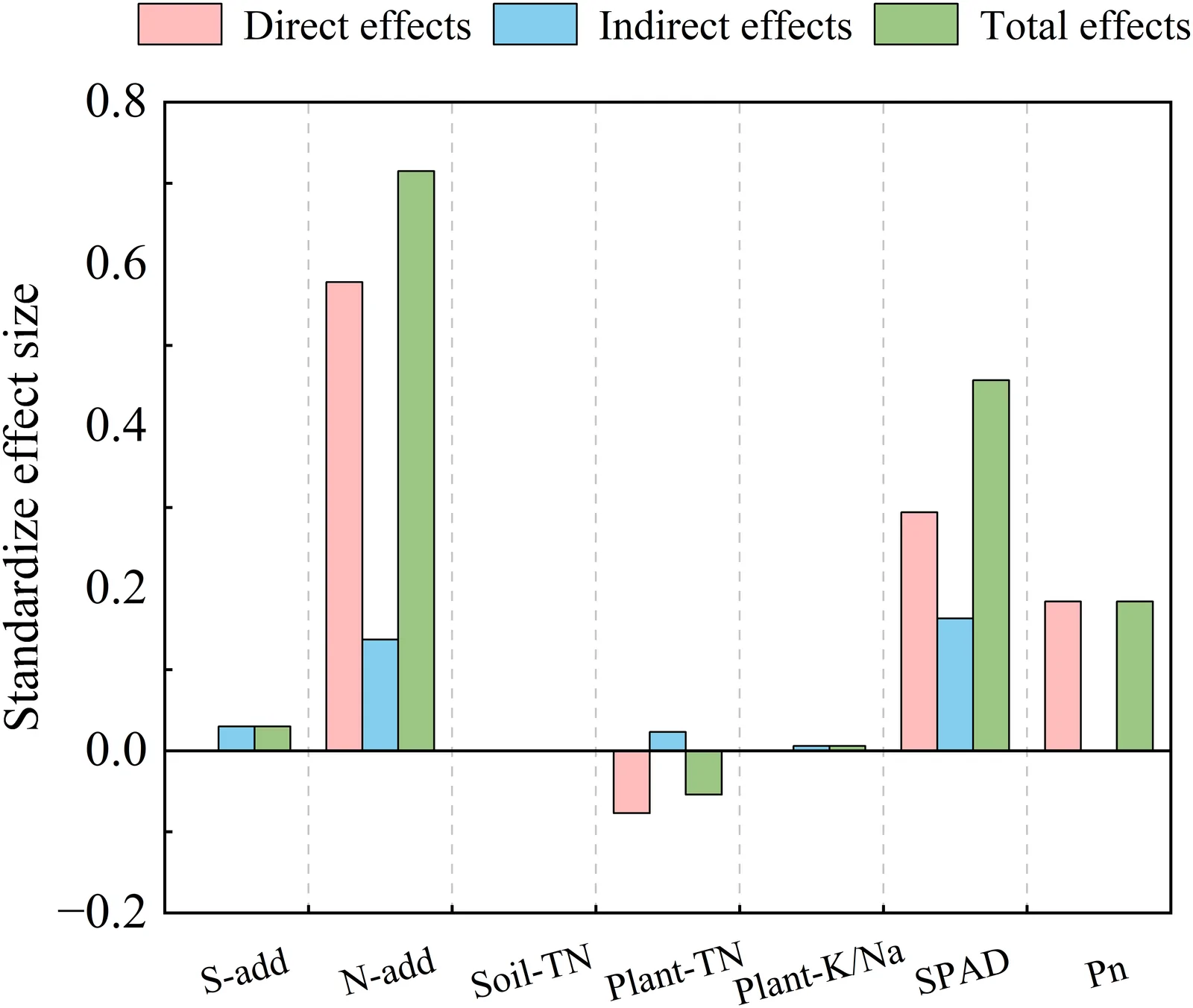
Total effects of the structural equation model.

Total effect analysis showed that (**Figure 15**) N-add had the largest total effect on growth, with a value of 0.715. Nitrogen addition promoted growth primarily through direct pathways, while also indirectly driving growth by increasing soil nitrogen, optimizing the K/Na ratio, and enhancing photosynthesis. The total effect of S-add was relatively weak; salt itself neither significantly inhibited nor promoted growth, and its effect was mainly manifested through interaction with nitrogen. The total effect of SPAD was 0.457; chlorophyll content directly drove growth and also indirectly promoted growth by enhancing photosynthesis. The total effect of *P*_n_ was 0.184; photosynthetic capacity was a direct physiological factor limiting growth. The total effect of Plant-K/Na was only 0.006, with a direct effect of 0 and an indirect effect of 0.006, indicating that the K/Na ratio was not a key factor driving growth, and its weak indirect effect on growth might be achieved through pathways such as influencing photosynthesis. The total effect of Plant-TN was -0.054, suggesting that excessively high plant nitrogen might produce a slight negative feedback. Nitrogen addition was the primary exogenous factor driving the growth of *L. bicolor*, while SPAD and *P*_n_ were the core physiological mediating variables.

## 4 Discussion

### 4.1 Effects of salt–nitrogen addition on the growth of *L. bicolor*

Plant growth and development serve as the most intuitive indicators for assessing responses to environmental stress, with morphological establishment and biomass accumulation directly reflecting plant adaptability to environmental conditions (Wang et al., 2024). In this study, *L. bicolor* grew extremely slowly under the S_0_N_0_ treatment; this was not a manifestation of stress-induced mortality, but rather a state of survival-growth under low-nutrient stress. Zhu et al. (2024) reported that appropriate concentrations of NaCl can promote the growth of *L. bicolor* by activating the synthesis of osmotic regulatory substances. As a typical recretohalophyte, *L. bicolor* lacks Na^+^ as an osmotic regulator in non-saline environments, resulting in inhibited growth. Under low- and moderate-salinity gradients, however, the growth of *L. bicolor* was promoted, exhibiting a pronounced halophilous effect.

Nitrogen, as an essential macronutrient for plant growth, plays a central role in alleviating salt stress and promoting biomass accumulation (Silva et al., 2024; Zhang et al., 2026). Zhang et al. (2026) pointed out that nitrogen not only serves as a nutrient element but also acts as a signaling molecule, coordinately regulating plant growth and stress tolerance through conserved pathways involving NLP transcription factors and the TOR/SnRK module. Zhang et al. (2023) found that short-term nitrogen addition enhanced the salt-uptake capacity of halophytes in the Yellow River Delta, indirectly facilitating plant adaptation to saline environments via plant–microbe interactions. In the present experiment, the 0.6% salinity and high-nitrogen treatment (S_2_N_45_) resulted in significant increases in plant height, stem diameter, root length, and biomass compared with S_0_N_0_, S_0_N_45_, and S_2_N_0_. Notably, the increments in aboveground and belowground dry weights under S_2_N_45_ were substantial, reflecting a functional convergence response of *L. bicolor* from suboptimal to favorable habitats. This also suggests that nitrogen addition may have not only enhanced nutrient uptake but also broadened the salt tolerance threshold of *L. bicolor*, indicating a synergistic promoting effect of salt–nitrogen interaction on plant growth. However, no significant differences were detected between the S_2_N_30_ and S_2_N_45_ treatments in terms of plant height, root length, or belowground dry weight, suggesting that excessive nitrogen addition may have induced a nitrogen saturation-like response in *L. bicolor*. When nitrogen supply exceeds plant demand, additional nitrogen input may no longer promote further growth, resulting in a plateau phase in plant response. Under nitrogen-free conditions, *L. bicolor* tended to increase its root-to-shoot ratio to acquire limited resources, whereas nitrogen addition effectively alleviated this response, leading to a more balanced biomass allocation—a finding consistent with the results of Yue et al (Yue et al., 2021).

### 4.2 Effects of salt–nitrogen addition on photosynthetic characteristics of *L. bicolor*

Photosynthesis serves as the central hub linking environmental nutrient supply and plant carbon accumulation. In this study, the response of *P*_n_ in *L. bicolor* was highly consistent with that of growth indices. Under the S_2_N_30_ treatment, *P*_n_ was significantly increased by 65.8% compared with the S_2_N_0_ treatment, indicating that nitrogen addition could markedly enhance the photosynthetic adaptation threshold of *L. bicolor* under saline conditions (Paula-Marinho et al., 2025). Jia et al. (2025) reported that irrigation amount, nitrogen, and salinity significantly affected leaf photosynthetic rates in *Arundo donax*, with a significant interaction between salinity and nitrogen. Paula-Marinho et al. (2025) further confirmed that NH_4_^+^ nutrition alleviated salt-induced damage to the photosynthetic system by maintaining PSII efficiency and enhancing CO_2_ assimilation rates. These findings are consistent with the mechanism of salt–nitrogen interaction promoting photosynthesis observed in our study.

Stomatal limitation and non-stomatal limitation are key factors affecting photosynthetic rates. In this study, *G*_s_ and *L*_s_ increased with increasing nitrogen addition, while *C*_i_ decreased significantly. This pattern is characteristic of typical non-stomatal limitation (Zhu et al., 2024), suggesting that under salt–nitrogen interaction, the enhancement of photosynthesis in *L. bicolor* leaves may primarily originate from improvements in non-stomatal factors, i.e., nitrogen addition enhanced the photosynthetic carbon assimilation capacity of mesophyll cells, allowing CO_2_ entering the intercellular spaces to be rapidly fixed, thereby reducing *C*_i_. Li et al. (2025) found that under 200 mM NaCl treatment, key carbon fixation genes such as Rubisco were upregulated in *S. salsa* leaves, and the lncRNA TCONS_00024624 enhanced photosynthetic capacity by regulating Rubisco activity. This molecular-level evidence provides cross-validation for the inference of non-stomatal limitation based on gas exchange parameters in this study, suggesting that the enhanced carbon assimilation capacity of halophytes under saline conditions may have a universal molecular basis. In the present study, WUE under the S_2_N_45_ treatment was significantly improved compared with the S_0_N_0_ and S_2_N_0_ treatments. Combining these results, it is inferred that nitrogen may indirectly promote moderate stomatal opening by optimizing plant water status and alleviating abscisic acid (ABA)-mediated stomatal signaling (Zhu et al., 2024).

Combined with the results of photosynthetic pigment content measurements (**Table 1**), SPAD value, Chl a, Chl b, and Chl a+b content all increased significantly with nitrogen addition and were enhanced under the S_2_N_30_ or S_2_N_45_ treatments, indicating that nitrogen addition promoted photosynthetic pigment synthesis and enhanced the light energy capture and conversion capacity of leaves. Tang et al. (2021) also confirmed in experiments with *Solanum nigrum* that nitrogen application increased Chl a, Chl b, and carotenoid contents, along with improved PSII photochemical efficiency. Furthermore, Car are important photoprotective substances in plants; in this study, their content under the S_2_N_30_ treatment was significantly higher than that under S_2_N_0_ and S_0_N_30_. Maslova et al. (2021) pointed out that Car not only function as accessory pigments in light energy absorption but also act as antioxidants protecting chlorophyll molecules from photo-oxidative damage. The concomitant accumulation of Car under moderate salt–nitrogen combined application suggests that nitrogen addition not only enhanced light-harvesting efficiency but also reinforced the photooxidative defense capacity of the photosynthetic apparatus. This dual effect may represent an additional important mechanism underlying the salt–nitrogen interaction-induced promotion of photosynthesis. Concurrently, the photosynthetic traits of *L. bicolor* exhibited a similar nitrogen saturation response, indicating that once plant nitrogen demand is met, further increases in nitrogen input do not enhance photosynthetic capacity.

### 4.3 Effects of salt–nitrogen addition on elemental contents and ecological stoichiometric characteristics of *L. bicolor*

Plant elemental contents and ecological stoichiometric characteristics can reflect nutrient utilization strategies, growth rates, and adaptability to environmental conditions (Zhao et al., 2023b). In this study, TN contents in both aboveground and belowground parts of *L. bicolor* increased with increasing salinity and nitrogen addition, whereas TP content exhibited a decreasing trend, accompanied by a decline in C:N ratio and an increase in N:P ratio. The observed rise in plant N but decline in P indicates that salt–nitrogen interaction altered the nutrient limitation pattern of *L. bicolor*. Nitrogen addition effectively alleviated the pre-existing nitrogen limitation under saline-alkali conditions. Meanwhile, saline conditions promoted nitrogen uptake by *L. bicolor*, which can be attributed to the fact that halophytes tend to accumulate more nitrogen-containing osmotic regulators (e.g., proline) under salt stress to maintain cell turgor (Ahanger et al., 2019; Zhao et al., 2020).On the other hand, nitrogen-induced rapid biomass growth exerts a dilution effect on tissue phosphorus concentration (Zuo et al., 2023). According to the N:P threshold hypothesis proposed by Güsewell (2004), the sustained increase in the N:P ratio observed in this study indicates that long-term nitrogen input may drive a shift from nitrogen limitation to phosphorus limitation in this ecosystem. This trend was further corroborated by soil stoichiometric characteristics (**Figure 12**), suggesting that coordinated nitrogen and phosphorus management should be given due attention in ecological restoration efforts in the Yellow River Delta.

Halophytes often adjust their K/Na ratio to optimize ion partitioning and energy utilization (Cheema et al., 2025). In this study, the aboveground and belowground K/Na ratios under the S_0_ treatment were generally higher than those under the S_2_ treatment. This does not indicate salt injury, but rather reflects a strategy of *L. bicolor* to actively utilize Na^+^ as a cheap osmotic regulator sequestered in vacuoles, thereby reducing dependence on K^+^ and lowering the cost of maintaining ion homeostasis (Ellouzi Hasna, 2023). Under the S_2_ condition, nitrogen addition treatments (N_15_–N_45_) exerted a more pronounced regulatory effect on the K/Na ratio in roots than in shoots. This may be attributed to competitive inhibition of Na^+^ by nitrogen at root absorption sites, which enhanced root K^+^ retention and Na^+^ efflux, thereby effectively preserving root ion selectivity (Javed et al., 2024). In contrast, the K/Na ratio in aboveground tissues did not show a corresponding increase under nitrogen addition, indicating that nitrogen-mediated regulation of ion homeostasis is tissue-specific—that is, it prioritizes the maintenance of root function while allowing aboveground parts to retain Na^+^ for osmotic adjustment.

### 4.4 Effects of salt–nitrogen addition on soil physicochemical properties

Foliar nitrogen input, together with plant growth feedback and minor nitrogen incorporation into the soil, induced changes in soil physicochemical properties. In this study, salinity addition increased soil pH and EC, while nitrogen addition had a limited direct effect on soil salinity. However, the salt–nitrogen interaction exerted significant regulatory effects on pH and EC. Unlike conventional nitrogen fertilization in agricultural soils, which typically induces acidification, soil pH in this study exhibited an increasing trend with nitrogen addition. This phenomenon may be attributed to nitrogen transformation processes (e.g., denitrification) that generated alkaline substances (Bai et al., 2023). You et al. (2025) also found that salt–nitrogen interaction significantly affected soil EC and pH, with EC being a key factor regulating rhizosphere microbial communities. In this study, although soil TN increased significantly under N addition, NO_3_^−^-N accumulation was suppressed by salinity, whereas NH_4_^+^-N accumulation increased markedly. This phenomenon may be attributable to the inhibitory effect of salinity on nitrifier activity (Zhou et al., 2021; Zhu et al., 2021). Under the same salinity level, soil NH_4_^+^-N content exhibited an increasing trend with rising nitrogen addition rates. This may be partly attributed to the inadvertent introduction of small amounts of urea solution into the soil during foliar spraying, which was subsequently hydrolyzed by soil urease into NH_4_^+^-N and then retained by soil particles or immobilized by microorganisms (Yu et al., 2016), thereby directly replenishing the soil ammonium pool. The remaining explanation may involve salt–nitrogen interactions indirectly modulating the rhizosphere microenvironment through their effects on plant growth. Under the S_0_N_45_, S_1_N_45_, and S_2_N_45_ treatments, MBC was significantly higher than that under the corresponding N_0_ treatments within the same salinity gradients. This indicates that sufficient nitrogen addition offset the inhibitory effect of 0.6% salinity on soil microorganisms. The stimulating effects of salt–nitrogen interaction on SOM and MBC (**Figure 10**) further support the plant–soil feedback mechanism: nitrogen addition promotes plant growth, increases root exudate inputs, and provides carbon sources for rhizosphere microorganisms, thereby alleviating the inhibitory effect of 0.6% salinity on microbial activity and facilitating the turnover of soil carbon and nitrogen pools (Bolan et al., 2025).

Under the S_2_N_30_ and S_2_N_45_ treatments, soil C:P and N:P ratios were elevated, indicating that nitrogen addition was disrupting the original nutrient balance and driving the soil nutrient structure from a low-nitrogen state toward a high-nitrogen and low-phosphorus state. The soil C:N ratio under the N_45_ treatment was higher than that under N_0_, suggesting that carbon accumulation outpaced nitrogen mineralization (Han et al., 2021). The shift in soil N:P ratio was consistent with the increasing trend in plant N:P ratio (**Figure 5**), implying that exogenous nitrogen input may alter soil stoichiometric relationships under the present experimental conditions; however, the long-term ecological effects still require further validation through sustained soil nitrogen input experiments. This coordinated shift in plant–soil stoichiometry further corroborates the potential risk of a transition from nitrogen limitation to phosphorus limitation under salt–nitrogen interaction, and provides a theoretical basis for integrated nitrogen and phosphorus management in ecological restoration of the Yellow River Delta.

## 5 Conclusion

Appropriate salt–nitrogen addition (0.6% salinity, 30–45 kg N·ha^−1^·yr^−1^) exerted significant synergistic promoting effects on the growth and photosynthesis of *L. bicolor*, with nitrogen addition being the primary exogenous factor driving plant growth. Under the S_2_N_45_ treatment, plant height, biomass, *P*_n_, WUE, and chlorophyll content all reached optimal levels, and a saturation effect of nitrogen addition was observed. Under salt–nitrogen interaction, *L. bicolor* enhanced photosynthetic efficiency through non-stomatal limitation mechanisms. Nitrogen addition optimized root ion homeostasis under 0.6% salinity conditions. Salt significantly affected soil pH, EC, and SOM; Foliar nitrogen input, together with plant–soil feedback processes, altered certain soil nutrient indices, with soil TN, NO_3_^−^-N, and NH_4_^+^-N contents showing significant responses. Under the conditions of this experiment, continuous foliar nitrogen input may alter plant–soil nutrient balance and potentially increase the risk of phosphorus limitation, highlighting the need for integrated nitrogen and phosphorus management in future ecological restoration efforts.

## Data Availability

The original contributions presented in the study are included in the article/supplementary material. Further inquiries can be directed to the corresponding author.
